# Supplementary material for: Rehabilitation and violence-related traumatic brain injury: A scoping review
Source: PLoS One. 2024 Nov 14;19(11):e0310803. doi: 10.1371/journal.pone.0310803 (PMC11563373; doi:10.1371/journal.pone.0310803)
Supplement: S1 File — (PDF) [file pone.0310803.s002.pdf]

Supplementary File 1

Search Strategy

Database: Ovid MEDLINE(R) ALL <1946 to January 10, 2023>
Platform: Ovid
Date searched: 2023-01-11

----

1 exp Violence/
2 (violence or violent or violently).tw,kf.
3 (assault*2 or assaultive).tw,kf.
4 (aggression or aggressor*).tw,kf.
5 (terrorism or terrorist?).tw,kf.
6 (rape? or raping).tw,kf.
7 (torture? or torturing).tw,kf.
8 "intentional injur*".tw,kf.
9 gunshot?.tw,kf.
10 (attacked or attacking).tw,kf.
11 (fight? or fighting).tw,kf.
12 ((psycholog* or sexual or physical* or emotion*) adj2 (neglect* or deprivat* or harm* or abus*)).tw,kf.
13 ((child* or wom#n or spous* or elder* or parent*) adj2 abus*).tw,kf.
14 (abusive or abused).tw,kf.
15 maldevelopment.tw,kf.
16 or/1-15 [Violence]
17 "Physical and Rehabilitation Medicine"/
18 exp rehabilitation/
19 rehab*.tw,kf,jw.
20 telerehab*.tw,kf,jw.
21 neurorehab*.tw,kf,jw.
22 rh.fs.
23 Rehabilitation Centers/
24 (physiatrist? or physiatry).tw,kf.
25 occupational therapy/
26 (occupational adj therap*).tw,kf,jw.
27 physical therapy specialty/
28 (physical adj therap*).tw,kf,jw.
29 physiotherap*.tw,kf,jw.
30 physio-therapist*.tw,kf,jw.
31 Speech-Language Pathology/
32 (speech adj2 (therap* or patholog*)).tw,kf,jw.
33 Neuropsychology/
34 Neuropsycholog*.tw,kf,jw.
35 Nutritionists/
36 (Nutritionist? or Dietician?).tw,kf,jw.
37 (therap* adj recreation*).tw,kf,jw.

38 child life specialist?.tw,kf.
39 play therapy/
40 (play adj therap*).tw,kf.
41 Respite Care/
42 respite.tw,kf.
43 Case Managers/
44 Case Management/
45 case manag*.tw,kf.
46 exp Social Work/
47 social work*.tw,kf,jw.
48 Forensic Nursing/
49 (nurse? or nursing).tw,kf,jw.
50 Community Integration/
51 (reintegrat* or re-integrat* or reentry or re-entry or resettle* or re-settle*).tw,kf.
52 Aftercare/
53 (Aftercare or "after care").tw,kf.
54 Transitional Care/
55 "transitional care".tw,kf.
56 or/17-55
57 exp Brain Injuries/
58 exp Brain Injuries, Traumatic/
59 exp Brain Concussion/
60 Craniocerebral Trauma/
61 tbi*2.tw,kf.
62 mtbi*2.tw,kf.
63 concuss*.tw,kf.
64 postconcuss*.tw,kf.
65 ((head* or brain* or cerebr* or crani* or skull* or intracran*) adj2 (injur* or trauma* or damag* or wound* or swell* or oedema* or edema* or fracture* or contusion* or pressur*)).tw,kf,jw.
66 ((brain* or cerebr* or intracerebr* or crani* or intracran* or head* or subdural* or epidural* or extradural* or retinol*) adj (haematoma* or hematoma* or hemorrhag* or haemorrhag* or bleed*)).tw,kf.
67 Shaken baby.tw,kf.
68 exp cognition disorders/
69 ((cogniti* or neurocogniti*) adj2 (impair* or dysfunction* or disorder* or declin*)).tw,kf.
70 or/57-69 [TBI/CI]
71 16 and 56 and 70
72 ((violen* or intentional or self-inflict* or other-inflict*) adj6 ((head* or brain* or cerebr* or crani* or skull* or intracran*) adj2 (injur* or trauma* or damag* or wound* or swell* or oedema* or edema* or fracture* or contusion* or pressur*))).tw,kf,jw.
73 ((violen* or intentional or self-inflict* or other-inflict*) adj6 ((brain* or cerebr* or intracerebr* or crani* or intracran* or head* or subdural* or epidural* or extradural*) adj (haematoma* or hematoma* or hemorrhag* or haemorrhag* or bleed*))).tw,kf.

74 ((violen* or intentional or self-inflict* or other-inflict*) adj6 (tbi*2 or mtbi*2 or concuss* or postconcuss*)).tw,kf.
75 72 or 73 or 74 [violence-induced TBI]
76 56 and 75
77 71 or 76
78 77 not (exp animals/ not humans.sh.)
79 limit 78 to english language
80 78 not 79

****

Database: Cochrane Central Register of Controlled Trials <December 2022>
Platform: Ovid
Date searched: 2023-01-11
----
1 exp Violence/
2 (violence or violent or violently).tw,kf.
3 (assault*2 or assaultive).tw,kf.
4 (aggression or aggressor*).tw,kf.
5 (terrorism or terrorist?).tw,kf.
6 (rape? or raping).tw,kf.
7 (torture? or torturing).tw,kf.
8 "intentional injur*".tw,kf.
9 gunshot?.tw,kf.
10 (attacked or attacking).tw,kf.
11 (fight? or fighting).tw,kf.
12 ((psycholog* or sexual or physical* or emotion*) adj2 (neglect* or deprivat* or harm* or abus*)).tw,kf.
13 ((child* or wom#n or spous* or elder* or parent*) adj2 abus*).tw,kf.
14 (abusive or abused).tw,kf.
15 maldevelopment.tw,kf.
16 or/1-15 [Violence]
17 "Physical and Rehabilitation Medicine"/
18 exp rehabilitation/
19 rehab*.tw,kf,jw.
20 telerehab*.tw,kf,jw.
21 neurorehab*.tw,kf,jw.
22 rh.fs.
23 Rehabilitation Centers/
24 (physiatrist? or physiatry).tw,kf.
25 occupational therapy/
26 (occupational adj therap*).tw,kf,jw.
27 physical therapy specialty/
28 (physical adj therap*).tw,kf,jw.
29 physiotherap*.tw,kf,jw.

30 physio-therapist*.tw,kf,jw.
31 Speech-Language Pathology/
32 (speech adj2 (therap* or patholog*)).tw,kf,jw.
33 Neuropsychology/
34 Neuropsycholog*.tw,kf,jw.
35 Nutritionists/
36 (Nutritionist? or Dietician?).tw,kf,jw.
37 (therap* adj recreation*).tw,kf,jw.
38 child life specialist?.tw,kf.
39 play therapy/
40 (play adj therap*).tw,kf.
41 Respite Care/
42 respite.tw,kf.
43 Case Managers/
44 Case Management/
45 case manag*.tw,kf.
46 exp Social Work/
47 social work*.tw,kf,jw.
48 Forensic Nursing/
49 (nurse? or nursing).tw,kf,jw.
50 Community Integration/
51 (reintegrat* or re-integrat* or reentry or re-entry or resettle* or re-settle*).tw,kf.
52 Aftercare/
53 (Aftercare or "after care").tw,kf.
54 Transitional Care/
55 "transitional care".tw,kf.
56 or/17-55
57 exp Brain Injuries/
58 exp Brain Injuries, Traumatic/
59 exp Brain Concussion/
60 Craniocerebral Trauma/
61 tbi*2.tw,kf.
62 mtbi*2.tw,kf.
63 concuss*.tw,kf.
64 postconcuss*.tw,kf.
65 ((head* or brain* or cerebr* or crani* or skull* or intracran*) adj2 (injur* or trauma* or damag* or wound* or swell* or oedema* or edema* or fracture* or contusion* or pressur*)).tw,kf,jw.
66 ((brain* or cerebr* or intracerebr* or crani* or intracran* or head* or subdural* or epidural* or extradural* or retinol*) adj (haematoma* or hematoma* or hemorrhag* or haemorrhag* or bleed*)).tw,kf.
67 Shaken baby.tw,kf.
68 exp cognition disorders/
69 ((cogniti* or neurocogniti*) adj2 (impair* or dysfunction* or disorder* or declin*)).tw,kf.
70 or/57-69 [TBI/CI]

71 16 and 56 and 70
72 ((violen* or intentional or self-inflict* or other-inflict*) adj6 ((head* or brain* or cerebr* or crani* or skull* or intracran*) adj2 (injur* or trauma* or damag* or wound* or swell* or oedema* or edema* or fracture* or contusion* or pressur*))).tw,kf,jw.
73 ((violen* or intentional or self-inflict* or other-inflict*) adj6 ((brain* or cerebr* or intracerebr* or crani* or intracran* or head* or subdural* or epidural* or extradural*) adj (haematoma* or hematoma* or hemorrhag* or haemorrhag* or bleed*))).tw,kf.
74 ((violen* or intentional or self-inflict* or other-inflict*) adj6 (tbi*2 or mtbi*2 or concuss* or postconcuss*)).tw,kf.
75 72 or 73 or 74 [violence-induced TBI]
76 56 and 75
77 71 or 76
78 77 not (exp animals/ not humans.sh.)
79 Trial registry record.pt.
80 78 not 79
81 limit 80 to english language
82 80 not 81

****

Database: APA PsycInfo <1806 to January Week 2 2023>
Platform: Ovid
Date searched: 2023-01-11
----
1 exp Violence/
2 (violence or violent or violently).ti,ab.
3 (assault*2 or assaultive).ti,ab.
4 (aggression or aggressor*).ti,ab.
5 (terrorism or terrorist?).ti,ab.
6 (rape? or raping).ti,ab.
7 (torture? or torturing).ti,ab.
8 "intentional injur*".ti,ab.
9 gunshot?.ti,ab.
10 (attacked or attacking).ti,ab.
11 (fight? or fighting).ti,ab.
12 ((psycholog* or sexual or physical* or emotion*) adj2 (neglect* or deprivat* or harm* or abus*)).ti,ab.
13 ((child* or wom#n or spous* or elder* or parent*) adj2 abus*).ti,ab.
14 (abusive or abused).ti,ab.
15 maldevelopment.ti,ab.
16 or/1-15
17 exp rehabilitation/
18 rehab*.ti,ab,jx.
19 telerehab*.ti,ab,jx.
20 neurorehab*.ti,ab,jx.
21 (physiatrist? or physiatry).ti,ab.

22 exp Rehabilitation Centers/
23 occupational therapy/
24 (occupational adj therap*).ti,ab,jx.
25 Physical therapy/
26 (physical adj therap*).ti,ab,jx.
27 physiotherap*.ti,ab,jx.
28 physio-therapist*.ti,ab,jx.
29 Speech Therapists/
30 (speech adj2 (therap* or patholog*)).ti,ab,jx.
31 Neuropsychology/
32 Neuropsycholog*.ti,ab,jx.
33 (Nutritionist? or Dietician?).ti,ab,jx.
34 (therap* adj recreation*).ti,ab,jx.
35 child life specialist?.ti,ab.
36 play therapy/
37 (play adj therap*).ti,ab.
38 Respite Care/
39 respite.ti,ab.
40 exp Social Casework/
41 Case Management/
42 case manag*.ti,ab.
43 exp Social Workers/
44 social work*.ti,ab,jx.
45 (nurse? or nursing).ti,ab,jx.
46 Reintegration/ or exp Social Integration/
47 (reintegrat* or re-integrat* or reentry or re-entry or resettle* or re-settle*).ti,ab.
48 Aftercare/
49 (Aftercare or "after care").ti,ab.
50 "transitional care".ti,ab.
51 or/17-50
52 exp Brain Injuries/
53 exp traumatic brain injury/
54 Brain concussion/
55 head injuries/
56 tbi*2.ti,ab.
57 mtbi*2.ti,ab.
58 concuss*.ti,ab.
59 postconcuss*.ti,ab.
60 ((head* or brain* or cerebr* or crani* or skull* or intracran*) adj2 (injur* or trauma* or damag* or wound* or swell* or oedema* or edema* or fracture* or contusion* or pressur*)).ti,ab,jx.
61 ((brain* or cerebr* or intracerebr* or crani* or intracran* or head* or subdural* or epidural* or extradural*) adj (haematoma* or hematoma* or hemorrhag* or haemorrhag* or bleed*)).ti,ab.
62 Cognitive Impairment/ or Mild Cognitive Impairment/

63 ((cogniti* or neurocogniti*) adj2 (impair* or dysfunction* or disorder* or declin*)).ti,ab.
64 or/52-63
65 16 and 51 and 64
66 ((violen* or intentional or self-inflict* or other-inflict*) adj6 ((head* or brain* or cerebr* or crani* or skull* or intracran*) adj2 (injur* or trauma* or damag* or wound* or swell* or oedema* or edema* or fracture* or contusion* or pressur*))).ti,ab,jx.
67 ((violen* or intentional or self-inflict* or other-inflict*) adj6 ((brain* or cerebr* or intracerebr* or crani* or intracran* or head* or subdural* or epidural* or extradural*) adj (haematoma* or hematoma* or hemorrhag* or haemorrhag* or bleed*))).ti,ab.
68 ((violen* or intentional or self-inflict* or other-inflict*) adj6 (tbi*2 or mtbi*2 or concuss* or postconcuss*)).ti,ab.
69 66 or 67 or 68
70 51 and 69
71 65 or 70
72 limit 71 to ("column/opinion" or dissertation or editorial)
73 71 not 72
74 limit 73 to animal
75 limit 74 to human
76 73 not (74 not 75)
77 limit 76 to english language
78 76 not 77

****

Database: Embase Classic+Embase <1947 to present>
Platform: Ovid
Date searched: 2023-01-11

----

1 exp Violence/
2 (violence or violent or violently).tw,kf.
3 (assault*2 or assaultive).tw,kf.
4 (aggression or aggressor*).tw,kf.
5 (terrorism or terrorist?).tw,kf.
6 (rape? or raping).tw,kf.
7 (torture? or torturing).tw,kf.
8 "intentional injur*".tw,kf.
9 gunshot?.tw,kf.
10 (attacked or attacking).tw,kf.
11 (fight? or fighting).tw,kf.
12 ((psycholog* or sexual or physical* or emotion*) adj2 (neglect* or deprivat* or harm* or abus*)).tw,kf.
13 ((child* or wom#n or spous* or elder* or parent*) adj2 abus*).tw,kf.
14 (abusive or abused).tw,kf.

15 maldevelopment.tw,kf.
16 or/1-15 [Violence]
17 rehabilitation medicine/ or physical medicine/
18 exp rehabilitation/
19 rehab*.tw,kf,jx.
20 telerehab*.tw,kf,jx.
21 neurorehab*.tw,kf,jx.
22 rh.fs.
23 Rehabilitation Center/
24 (physiatrist? or physiatry).tw,kf.
25 occupational therapy/
26 (occupational adj therap*).tw,kf,jx.
27 exp physiotherapy/
28 (physical adj therap*).tw,kf,jx.
29 physiotherap*.tw,kf,jx.
30 physio-therapist*.tw,kf,jx.
31 "speech and language rehabilitation"/
32 (speech adj2 (therap* or patholog*)).tw,kf,jx.
33 Neuropsychology/
34 Neuropsycholog*.tw,kf,jx.
35 Dietitian/
36 (Nutritionist? or Dietician?).tw,kf,jx.
37 (therap* adj recreation*).tw,kf,jx.
38 child life specialist?.tw,kf.
39 play therapy/
40 (play adj therap*).tw,kf.
41 Respite Care/
42 respite.tw,kf.
43 Case Manager/
44 Case Management/
45 case manag*.tw,kf.
46 Social Work/
47 social work*.tw,kf,jx.
48 Forensic Nursing/
49 (nurse? or nursing).tw,kf,jx.
50 Community Integration/ or community reintegration/
51 (reintegrat* or re-integrat* or reentry or re-entry or resettle* or re-settle*).tw,kf.
52 Aftercare/
53 (Aftercare or "after care").tw,kf.
54 Transitional Care/
55 "transitional care".tw,kf.
56 or/17-55
57 exp Brain Injury/
58 exp traumatic brain injury/
59 Brain concussion/ or postconcussion syndrome/
60 head injury/

61 tbi*2.tw,kf.
62 mtbi*2.tw,kf.
63 concuss*.tw,kf.
64 postconcuss*.tw,kf.
65 ((head* or brain* or cerebr* or crani* or skull* or intracran*) adj2 (injur* or trauma* or damag* or wound* or swell* or oedema* or edema* or fracture* or contusion* or pressur*)).tw,kf,jx.
66 ((brain* or cerebr* or intracerebr* or crani* or intracran* or head* or subdural* or epidural* or extradural* or retinol*) adj (haematoma* or hematoma* or hemorrhag* or haemorrhag* or bleed*)).tw,kf.
67 shaken baby syndrome/
68 Shaken baby.tw,kf.
69 exp cognitive defect/
70 ((cogniti* or neurocogniti*) adj2 (impair* or dysfunction* or disorder* or declin*)).tw,kf.
71 or/57-70 [TBI/CI]
72 16 and 56 and 71
73 ((violen* or intentional or self-inflict* or other-inflict*) adj6 ((head* or brain* or cerebr* or crani* or skull* or intracran*) adj2 (injur* or trauma* or damag* or wound* or swell* or oedema* or edema* or fracture* or contusion* or pressur*))).tw,kf,jx.
74 ((violen* or intentional or self-inflict* or other-inflict*) adj6 ((brain* or cerebr* or intracerebr* or crani* or intracran* or head* or subdural* or epidural* or extradural*) adj (haematoma* or hematoma* or hemorrhag* or haemorrhag* or bleed*))).tw,kf.
75 ((violen* or intentional or self-inflict* or other-inflict*) adj6 (tbi*2 or mtbi*2 or concuss* or postconcuss*)).tw,kf.
76 73 or 74 or 75 [violence-induced TBI]
77 56 and 76
78 72 or 77
79 78 not (((rat or rats or mouse or mice or swine or porcine or murine or sheep or lambs or pigs or piglets or rabbit or rabbits or cat or cats or dog or dogs or cattle or bovine or monkey or monkeys or trout or marmoset$1).ti. and animal experiment/) or (Animal experiment/ not (human experiment/ or human/)))
80 79 not medline.cr.
81 limit 80 to conference abstracts
82 80 not 81
83 limit 82 to english language
84 82 not 83

****

Database: CINAHL
Platform: EBSCO
Date searched: 2023-01-12

#	**Query**
S1	(MH "Violence+") OR (MH "Patient Assault") OR (MH "Patient Abuse") OR (MH "Student Abuse") OR (MH "Verbal Abuse") OR (MH "Emotional Abuse")
S2	TI (violence or violent or violently) OR AB (violence or violent or violently)
S3	TI ((assault*2 or assaultive)) OR AB ((assault*2 or assaultive))
S4	TI ((aggression or aggressor*)) OR AB ((aggression or aggressor*))
S5	TI (terrorism or terrorist#) OR AB (terrorism or terrorist#)
S6	TI ((rape# or raping)) OR AB ((rape# or raping))
S7	TI ((torture# or torturing)) OR ((torture# or torturing))
S8	TI "intentional injur*" OR AB "intentional injur*"
S9	TI gunshot# OR AB gunshot#
S10	TI ((attacked or attacking)) OR AB ((attacked or attacking))
S11	TI ((fight# or fighting)) OR AB ((fight# or fighting))
S12	TI (((psycholog* or sexual or physical* or emotion*) n2 (neglect* or deprivat* or harm* or abus*))) OR AB (((psycholog* or sexual or physical* or emotion*) n2 (neglect* or deprivat* or harm* or abus*)))
S13	TI (((child* or wom?n or spous* or elder* or parent*) n2 abus*)) OR AB (((child* or wom?n or spous* or elder* or parent*) n2 abus*))
S14	TI ((abusive or abused)) OR AB ((abusive or abused))
S15	TI maldevelopment OR AB maldevelopment
S16	S1 OR S2 OR S3 OR S4 OR S5 OR S6 OR S7 OR S8 OR S9 OR S10 OR S11 OR S12 OR S13 OR S14 OR S15
S17	(MH "Rehabilitation+")
S18	(MH "Physical Medicine")
S19	TI rehab* OR AB rehab* OR SO rehab*
S20	TI telerehab* OR AB telerehab* OR SO telerehab*
S21	TI neurorehab* OR AB neurorehab* OR SO neurorehab*
S22	TI ((physiatrist* or physiatry)) OR AB ((physiatrist* or physiatry))
S23	(MH "Rehabilitation Centers+")
S24	(MH "Occupational Therapy+") or (MH "Occupational Therapists")
S25	TI (occupational n1 therap*) OR AB (occupational n1 therap*) OR SO (occupational n1 therap*)
S26	(MH "Physical Therapy+") OR (MH "Physical Therapists")
S27	TI (physical n1 therap*) OR AB (physical n1 therap*) OR SO (physical n1 therap*)
S28	TI physiotherap* OR AB physiotherap* OR SO physiotherap*
S29	TI physio-therapist* OR AB physio-therapist* OR SO physio-therapist*
S30	(MH "Speech-Language Pathologists") OR (MH "Speech-Language Pathology Assistants")
S31	TI ((speech n2 (therap* or patholog*))) OR AB ((speech n2 (therap* or patholog*))) OR SO ((speech n2 (therap* or patholog*)))
S32	(MH "Neuropsychology")

S33	TI Neuropsycholog* OR AB Neuropsycholog* OR SO Neuropsycholog
S34	(MH "Dietitians")
S35	TI ((Nutritionist* or Dietician*)) OR AB ((Nutritionist* or Dietician*)) OR SO ((Nutritionist* or Dietician*))
S36	(MH "Recreational Therapy")
S37	(MH "Recreational Therapists")
S38	TI (therap* n1 recreation*) OR AB (therap* n1 recreation*) OR SO (therap* n1 recreation*)
S39	TI child life specialist* OR AB child life specialist* OR SO child life specialist*
S40	(MH "Play Therapy")
S41	TI (play n1 therap*) OR AB (play n1 therap*) OR (play n1 therap*)
S42	(MH "Respite Care")
S43	TI respite OR AB respite
S44	(MH "Case Managers")
S45	(MH "Case Management")
S46	TI "case manag*" OR AB "case manag*"
S47	(MH "Social Work+")
S48	(MH "Social Workers")
S49	TI "social work*" OR AB "social work*" OR SO "social work*"
S50	(MH "Forensic Nursing")
S51	TI ((nurse or nurses or nursing)) OR AB ((nurse or nurses or nursing)) OR SO ((nurse or nurses or nursing))
S52	(MH "Community Reintegration")
S53	TI ((reintegrat* or re-integrat* or reentry or re-entry or resettle* or re-settle)) OR AB ((reintegrat* or re-integrat* or reentry or re-entry or resettle* or re-settle))
S54	(MH "After Care")
S55	TI ((Aftercare or "after care")) OR AB ((Aftercare or "after care"))
S56	TI "transitional care" OR AB "transitional care"
S57	(MH "Transitional Care")
S58	S17 OR S18 OR S19 OR S20 OR S21 OR S22 OR S23 OR S24 OR S25 OR S26 OR S27 OR S28 OR S29 OR S30 OR S31 OR S32 OR S33 OR S34 OR S35 OR S36 OR S37 OR S38 OR S39 OR S40 OR S41 OR S42 OR S43 OR S44 OR S45 OR S46 OR S47 OR S48 OR S49 OR S50 OR S51 OR S52 OR S53 OR S54 OR S55 OR S56 OR S57
S59	(MH "Brain Injuries+")
S60	(MH "Head Injuries")
S61	(MH "Brain Concussion+")
S62	TI (TBI* OR mTBI*) OR AB (TBI* OR mTBI*)
S63	TI (concuss* or postconcuss*) OR AB (concuss* or postconcuss*)
S64	TI (((head* or brain* or cerebr* or crani* or skull* or intracran*) n2 (injur* or trauma* or damag* or wound* or swell* or oedema* or edema* or fracture* or contusion* or pressur*))) OR AB (((head* or brain* or cerebr* or crani* or skull* or intracran*) n2 (injur* or trauma* or damag* or wound* or swell* or oedema* or edema* or fracture* or contusion* or pressur*))) OR SO (((head* or brain* or

	cerebr* or crani* or skull* or intracran*) n2 (injur* or trauma* or damag* or wound* or swell* or oedema* or edema* or fracture* or contusion* or pressur*)))
S65	TI (((brain* or cerebr* or intracerebr* or crani* or intracran* or head* or subdural* or epidural* or extradural* or retinol*) n1 (haematoma* or hematoma* or hemorrhag* or haemorrhag* or bleed*))) OR AB (((brain* or cerebr* or intracerebr* or crani* or intracran* or head* or subdural* or epidural* or extradural* or retinol*) n1 (haematoma* or hematoma* or hemorrhag* or haemorrhag* or bleed*)))
S66	(MH "Cognition Disorders+")
S67	TI (((cogniti* or neurocogniti*) n2 (impair* or dysfunction* or disorder* or declin*))) OR AB (((cogniti* or neurocogniti*) n2 (impair* or dysfunction* or disorder* or declin*)))
S68	TI Shaken baby OR AB Shaken baby
S69	(MH "Shaken Baby Syndrome")
S70	S59 OR S60 OR S61 OR S62 OR S63 OR S64 OR S65 OR S66 OR S67 OR S68 OR S69
S71	S16 AND S58 AND S70
S72	TI (((violen* or intentional or self-inflict* or other-inflict*) n6 ((head* or brain* or cerebr* or crani* or skull* or intracran*) n2 (injur* or trauma* or damag* or wound* or swell* or oedema* or edema* or fracture* or contusion* or pressur*)))) OR AB (((violen* or intentional or self-inflict* or other-inflict*) n6 ((head* or brain* or cerebr* or crani* or skull* or intracran*) n2 (injur* or trauma* or damag* or wound* or swell* or oedema* or edema* or fracture* or contusion* or pressur*)))) OR SO (((violen* or intentional or self-inflict* or other-inflict*) n6 ((head* or brain* or cerebr* or crani* or skull* or intracran*) n2 (injur* or trauma* or damag* or wound* or swell* or oedema* or edema* or fracture* or contusion* or pressur*))))
S73	TI (((violen* or intentional or self-inflict* or other-inflict*) n6 ((brain* or cerebr* or intracerebr* or crani* or intracran* or head* or subdural* or epidural* or extradural*) n1 (haematoma* or hematoma* or hemorrhag* or haemorrhag* or bleed*)))) OR AB (((violen* or intentional or self-inflict* or other-inflict*) n6 ((brain* or cerebr* or intracerebr* or crani* or intracran* or head* or subdural* or epidural* or extradural*) n1 (haematoma* or hematoma* or hemorrhag* or haemorrhag* or bleed*)))) OR SO (((violen* or intentional or self-inflict* or other-inflict*) n6 ((brain* or cerebr* or intracerebr* or crani* or intracran* or head* or subdural* or epidural* or extradural*) n1 (haematoma* or hematoma* or hemorrhag* or haemorrhag* or bleed*))))
S74	TI (((violen* or intentional or self-inflict* or other-inflict*) n6 (tbi*2 or mtbi*2 or concuss* or postconcuss*))) OR AB (((violen* or intentional or self-inflict* or other-inflict*) n6 (tbi*2 or mtbi*2 or concuss* or postconcuss*))) OR SO (((violen* or intentional or self-inflict* or other-inflict*) n6 (tbi*2 or mtbi*2 or concuss* or postconcuss*)))
S75	S72 OR S73 OR S74
S76	S58 AND S75
S77	S71 OR S76
S78	S77
S79	S77 NOT S78

****

Database: Criminal Justice Abstracts
Platform: EBSCO
Date searched: 2023-01-12

#	**Query**
S1	((((ZU "violence")) or ((ZU "physical abuse"))) or ((ZU "assault & battery"))) or ((ZU "aggression (psychology)"))
S2	TI (violence or violent or violently) OR AB (violence or violent or violently)
S3	TI ((assault*2 or assaultive)) OR AB ((assault*2 or assaultive))
S4	TI ((aggression or aggressor*)) OR AB ((aggression or aggressor*))
S5	TI (terrorism or terrorist#) OR AB (terrorism or terrorist#)
S6	TI ((rape# or raping)) OR AB ((rape# or raping))
S7	TI ((torture# or torturing)) OR ((torture# or torturing))
S8	TI "intentional injur*" OR AB "intentional injur*"
S9	TI gunshot# OR AB gunshot#
S10	TI ((attacked or attacking)) OR AB ((attacked or attacking))
S11	TI ((fight# or fighting)) OR AB ((fight# or fighting))
S12	TI (((psycholog* or sexual or physical* or emotion*) n2 (neglect* or deprivat* or harm* or abus*))) OR AB (((psycholog* or sexual or physical* or emotion*) n2 (neglect* or deprivat* or harm* or abus*)))
S13	TI (((child* or wom?n or spous* or elder* or parent*) n2 abus*)) OR AB (((child* or wom?n or spous* or elder* or parent*) n2 abus*))
S14	TI ((abusive or abused)) OR AB ((abusive or abused))
S15	TI maldevelopment OR AB maldevelopment
S16	S1 OR S2 OR S3 OR S4 OR S5 OR S6 OR S7 OR S8 OR S9 OR S10 OR S11 OR S12 OR S13 OR S14 OR S15
S17	(((((((((((((ZU "rehabilitation")) or ((ZU "physical medicine"))) or ((ZU "rehabilitation centers"))) or ((ZU "occupational therapy") or (ZU "occupational therapy assistants"))) or ((ZU "physical therapy"))) or ((ZU "neuropsychology"))) or ((ZU "nutritionists"))) or ((ZU "dietitians"))) or ((ZU "recreational therapy"))) or ((ZU "play therapy"))) or ((ZU "respite care"))) or ((ZU "forensic nursing"))) or ((ZU "transitional care"))
S18	TI rehab* OR AB rehab* OR SO rehab*
S19	TI telerehab* OR AB telerehab* OR SO telerehab*
S20	TI neurorehab* OR AB neurorehab* OR SO neurorehab*
S21	TI ((physiatrist* or physiatry)) OR AB ((physiatrist* or physiatry))
S22	TI (occupational n1 therap*) OR AB (occupational n1 therap*) OR SO (occupational n1 therap*)
S23	TI (physical n1 therap*) OR AB (physical n1 therap*) OR SO (physical n1 therap*)
S24	TI physiotherap* OR AB physiotherap* OR SO physiotherap*
S25	TI physio-therapist* OR AB physio-therapist* OR SO physio-therapist*
S26	TI ((speech n2 (therap* or patholog*))) OR AB ((speech n2 (therap* or patholog*))) OR SO ((speech n2 (therap* or patholog*)))
S27	TI Neuropsycholog* OR AB Neuropsycholog* OR SO Neuropsycholog

S28	TI ((Nutritionist* or Dietician*)) OR AB ((Nutritionist* or Dietician*)) OR SO ((Nutritionist* or Dietician*))
S29	TI (therap* n1 recreation*) OR AB (therap* n1 recreation*) OR SO (therap* n1 recreation*)
S30	TI child life specialist* OR AB child life specialist* OR SO child life specialist*
S31	TI (play n1 therap*) OR AB (play n1 therap*) OR (play n1 therap*)
S32	TI respite OR AB respite
S33	TI "case manag*" OR AB "case manag*"
S34	TI "social work*" OR AB "social work*" OR SO "social work*"
S35	TI ((nurse or nurses or nursing)) OR AB ((nurse or nurses or nursing)) OR SO ((nurse or nurses or nursing))
S36	TI ((reintegrat* or re-integrat* or reentry or re-entry or resettle* or re-settle)) OR AB ((reintegrat* or re-integrat* or reentry or re-entry or resettle* or re-settle))
S37	TI ((Aftercare or "after care")) OR AB ((Aftercare or "after care"))
S38	TI "transitional care" OR AB "transitional care"
S39	S17 OR S18 OR S19 OR S20 OR S21 OR S22 OR S23 OR S24 OR S25 OR S26 OR S27 OR S28 OR S29 OR S30 OR S31 OR S32 OR S33 OR S34 OR S35 OR S36 OR S37 OR S38
S40	(((ZU "head injuries"))) or ((ZU "brain injuries"))) or ((ZU "cognition disorders"))
S41	TI (TBI* OR mTBI*) OR AB (TBI* OR mTBI*)
S42	TI (concuss* or postconcuss*) OR AB (concuss* or postconcuss*)
S43	TI (((head* or brain* or cerebr* or crani* or skull* or intracran*) n2 (injur* or trauma* or damag* or wound* or swell* or oedema* or edema* or fracture* or contusion* or pressur*))) OR AB (((head* or brain* or cerebr* or crani* or skull* or intracran*) n2 (injur* or trauma* or damag* or wound* or swell* or oedema* or edema* or fracture* or contusion* or pressur*))) OR SO (((head* or brain* or cerebr* or crani* or skull* or intracran*) n2 (injur* or trauma* or damag* or wound* or swell* or oedema* or edema* or fracture* or contusion* or pressur*)))
S44	TI (((brain* or cerebr* or intracerebr* or crani* or intracran* or head* or subdural* or epidural* or extradural* or retinol*) n1 (haematoma* or hematoma* or hemorrhag* or haemorrhag* or bleed*))) OR AB (((brain* or cerebr* or intracerebr* or crani* or intracran* or head* or subdural* or epidural* or extradural* or retinol*) n1 (haematoma* or hematoma* or hemorrhag* or haemorrhag* or bleed*)))
S45	TI (((cogniti* or neurocogniti*) n2 (impair* or dysfunction* or disorder* or declin*))) OR AB (((cogniti* or neurocogniti*) n2 (impair* or dysfunction* or disorder* or declin*)))
S46	TI Shaken baby OR AB Shaken baby
S47	(ZU "shaken baby syndrome")
S48	S40 OR S41 OR S42 OR S43 OR S44 OR S45 OR S46
S49	S16 AND S39 AND S48
S50	TI (((violen* or intentional or self-inflict* or other-inflict*) n6 ((head* or brain* or cerebr* or crani* or skull* or intracran*) n2 (injur* or trauma* or damag* or wound* or swell* or oedema* or edema* or fracture* or contusion* or pressur*)))) OR AB (((violen* or intentional or self-inflict* or other-inflict*) n6 ((head* or brain* or cerebr* or crani* or skull* or intracran*) n2 (injur* or trauma* or damag* or wound*

	or swell* or oedema* or edema* or fracture* or contusion* or pressur*)))) OR SO (((violen* or intentional or self-inflict* or other-inflict*) n6 ((head* or brain* or cerebr* or crani* or skull* or intracran*) n2 (injur* or trauma* or damag* or wound* or swell* or oedema* or edema* or fracture* or contusion* or pressur*))))
S51	TI (((violen* or intentional or self-inflict* or other-inflict*) n6 ((brain* or cerebr* or intracerebr* or crani* or intracran* or head* or subdural* or epidural* or extradural*) n1 (haematoma* or hematoma* or hemorrhag* or haemorrhag* or bleed*)))) OR AB (((violen* or intentional or self-inflict* or other-inflict*) n6 ((brain* or cerebr* or intracerebr* or crani* or intracran* or head* or subdural* or epidural* or extradural*) n1 (haematoma* or hematoma* or hemorrhag* or haemorrhag* or bleed*)))) OR SO (((violen* or intentional or self-inflict* or other-inflict*) n6 ((brain* or cerebr* or intracerebr* or crani* or intracran* or head* or subdural* or epidural* or extradural*) n1 (haematoma* or hematoma* or hemorrhag* or haemorrhag* or bleed*))))
S52	TI (((violen* or intentional or self-inflict* or other-inflict*) n6 (tbi*2 or mtbi*2 or concuss* or postconcuss*))) OR AB (((violen* or intentional or self-inflict* or other-inflict*) n6 (tbi*2 or mtbi*2 or concuss* or postconcuss*))) OR SO (((violen* or intentional or self-inflict* or other-inflict*) n6 (tbi*2 or mtbi*2 or concuss* or postconcuss*)))
S53	S50 OR S51 OR S52
S54	S39 AND S53
S55	S49 OR S54
S56	S55
S57	S55 NOT S56

****

Database: Applied Social Sciences Index & Abstracts (ASSIA)
Platform: Proquest
Date searched: 2023-01-12

S1	(NOFT(violence OR violent OR violently OR assault*2 OR assaultive OR aggression OR aggressor*) OR NOFT(terrorism OR terrorist* OR rape OR raped OR rapes OR raping) OR NOFT(torture* or torturing OR gunshot* OR attacked OR attacking OR fight OR fights OR fighting) OR NOFT((child* OR women OR woman OR spous* OR elder* OR parent*) n/2 abus*) OR NOFT(maldevelopment)) AND (NOFT(TBI* OR mTBI* OR concuss* OR postconcuss*) OR NOFT((head* or brain* or cerebr* or crani* or skull* or intracran*) n/2 (injur* or trauma* or damag* or wound* or swell* or oedema* or edema* or fracture* or contusion* or pressur*)) OR PUB((head* or brain* or cerebr* or crani* or skull* or intracran*) n/2 (injur* or trauma* or damag* or wound* or swell* or oedema* or edema* or fracture* or contusion* or pressur*)) OR NOFT((brain* or cerebr* or intracerebr* or crani* or intracran* or head* or subdural* or epidural* or extradural* or retinol*) n/1 (haematoma* or hematoma* or hemorrhag* or haemorrhag* or bleed*)) OR NOFT((cogniti* or neurocogniti*)

	n/2 (impair* or dysfunction* or disorder* or declin*)) OR NOFT(shaken baby)) AND (NOFT(rehab* OR telerehab* OR neurorehab* OR physiatrist* OR physiatry OR Neuropsycholog* OR Nutritionist* or Dietician* OR respite) OR PUB(rehab* OR telerehab* OR neurorehab* OR Neuropsycholog* OR Nutritionist* or Dietician* OR Respite) OR NOFT(occupational n/1 therap*) OR PUB(occupational n/1 therap*) OR NOFT(physical n1 therap*) OR PUB(physical n/1 therap*) OR NOFT(physiotherap* or "physio-therap*") OR PUB(physiotherap* or "physio-therap*") OR NOFT(speech n/2 (therap* or patholog*)) OR PUB(speech n/2 (therap* or patholog*)) OR NOFT(therap* n/1 recreation*) OR PUB(therap* n/1 recreation*) OR NOFT("child life specialist*") OR NOFT(play n/1 therap*) OR NOFT("case manag*") OR NOFT("social work*") OR NOFT(nurse or nurses or nursing) Or NOFT(reintegrat* or "re-integrat*" or reentry or "re-entry" or resettle* or "re-settle") OR NOFT(Aftercare or "after care") OR NOFT("transitional care"))
S2	(NOFT((psycholog* OR sexual OR physical* OR emotion*) n/2 (harm* or deprivat*)) OR NOFT((psycholog* OR sexual OR physical* OR emotion*) n/2 (abus*))) AND (NOFT(TBI* OR mTBI* OR concuss* OR postconcuss*) OR NOFT((head* or brain* or cerebr* or crani* or skull* or intracran*) n/2 (injur* or trauma* or damag* or wound* or swell* or oedema* or edema* or fracture* or contusion* or pressur*)) OR PUB((head* or brain* or cerebr* or crani* or skull* or intracran*) n/2 (injur* or trauma* or damag* or wound* or swell* or oedema* or edema* or fracture* or contusion* or pressur*)) OR NOFT((brain* or cerebr* or intracerebr* or crani* or intracran* or head* or subdural* or epidural* or extradural* or retinol*) n/1 (haematoma* or hematoma* or hemorrhag* or haemorrhag* or bleed*)) OR NOFT((cogniti* or neurocogniti*) n/2 (impair* or dysfunction* or disorder* or declin*)) OR NOFT(shaken baby)) AND (NOFT(rehab* OR telerehab* OR neurorehab* OR physiatrist* OR physiatry OR Neuropsycholog* OR Nutritionist* or Dietician* OR respite) OR PUB(rehab* OR telerehab* OR neurorehab* OR Neuropsycholog* OR Nutritionist* or Dietician* OR Respite) OR NOFT(occupational n/1 therap*) OR PUB(occupational n/1 therap*) OR NOFT(physical n1 therap*) OR PUB(physical n/1 therap*) OR NOFT(physiotherap* or "physio-therap*") OR PUB(physiotherap* or "physio-therap*") OR NOFT(speech n/2 (therap* or patholog*)) OR PUB(speech n/2 (therap* or patholog*)) OR NOFT(therap* n/1 recreation*) OR PUB(therap* n/1 recreation*) OR NOFT("child life specialist*") OR NOFT(play n/1 therap*) OR NOFT("case manag*") OR NOFT("social work*") OR NOFT(nurse or nurses or nursing) Or NOFT(reintegrat* or "re-integrat*" or reentry or "re-entry" or resettle* or "re-settle") OR NOFT(Aftercare or "after care") OR NOFT("transitional care"))
S3	(NOFT(((violen* or intentional or self-inflict* or other-inflict*) n/6 (head* or brain* or cerebr* or crani* or skull* or intracran*)) n/2 (injur* or trauma* or damag* or wound* or swell* or oedema* or edema* or fracture* or contusion* or pressur*)) OR NOFT(((violen* or intentional or self-inflict* or other-inflict*) n/6 (brain* or cerebr* or intracerebr* or crani* or intracran* or head* or subdural* or epidural* or extradural*)) n/1 (haematoma* or hematoma* or hemorrhag* or

	haemorrhag* or bleed*)) OR NOFT((violen* or intentional or self-inflict* or other-inflict*) n/6 (tbi*2 or mtbi*2 or concuss* or postconcuss*))) AND (NOFT(rehab* OR telerehab* OR neurorehab* OR physiatrist* OR physiatry OR Neuropsycholog* OR Nutritionist* or Dietician* OR respite) OR PUB(rehab* OR telerehab* OR neurorehab* OR Neuropsycholog* OR Nutritionist* or Dietician* OR Respite) OR NOFT(occupational n/1 therap*) OR PUB(occupational n/1 therap*) OR NOFT(physical n1 therap*) OR PUB(physical n/1 therap*) OR NOFT(physiotherap* or "physio-therap*") OR PUB(physiotherap* or "physio-therap*") OR NOFT(speech n/2 (therap* or patholog*)) OR PUB(speech n/2 (therap* or patholog*)) OR NOFT(therap* n/1 recreation*) OR PUB(therap* n/1 recreation*) OR NOFT("child life specialist*") OR NOFT(play n/1 therap*) OR NOFT("case manag*") OR NOFT("social work*") OR NOFT(nurse or nurses or nursing) OR NOFT(reintegrat* or "re-integrat*" or reentry or "re-entry" or resettle* or "re-settle") OR NOFT(Aftercare or "after care") OR NOFT("transitional care"))
S4	S1 OR S2 OR S3

*All English

****

Database: Proquest Nursing and Allied Health
Platform: Proquest
Date searched: 2023-01-12

S1	(NOFT(violence OR violent OR violently OR assault*2 OR assaultive OR aggression OR aggressor*) OR NOFT(terrorism OR terrorist* OR rape OR raped OR rapes OR raping) OR NOFT(torture* or torturing OR gunshot* OR attacked OR attacking OR fight OR fights OR fighting) OR NOFT((child* OR women OR woman OR spous* OR elder* OR parent*) n/2 abus*) OR NOFT(maldevelopment)) AND (NOFT(TBI* OR mTBI* OR concuss* OR postconcuss*) OR NOFT((head* or brain* or cerebr* or crani* or skull* or intracran*) n/2 (injur* or trauma* or damag* or wound* or swell* or oedema* or edema* or fracture* or contusion* or pressur*)) OR PUB((head* or brain* or cerebr* or crani* or skull* or intracran*) n/2 (injur* or trauma* or damag* or wound* or swell* or oedema* or edema* or fracture* or contusion* or pressur*)) OR NOFT((brain* or cerebr* or intracerebr* or crani* or intracran* or head* or subdural* or epidural* or extradural* or retinol*) n/1 (haematoma* or hematoma* or hemorrhag* or haemorrhag* or bleed*)) OR NOFT((cogniti* or neurocogniti*) n/2 (impair* or dysfunction* or disorder* or declin*)) OR NOFT(shaken baby)) AND (NOFT(rehab* OR telerehab* OR neurorehab* OR physiatrist* OR physiatry OR Neuropsycholog* OR Nutritionist* or Dietician* OR respite) OR PUB(rehab* OR telerehab* OR neurorehab* OR Neuropsycholog* OR Nutritionist* or Dietician* OR Respite) OR NOFT(occupational n/1 therap*) OR

	PUB(occupational n/1 therap*) OR NOFT(physical n1 therap*) OR PUB(physical n/1 therap*) OR NOFT(physiotherap* or "physio-therap*") OR PUB(physiotherap* or "physio-therap*") OR NOFT(speech n/2 (therap* or patholog*)) OR PUB(speech n/2 (therap* or patholog*)) OR NOFT(therap* n/1 recreation*) OR PUB(therap* n/1 recreation*) OR NOFT("child life specialist*") OR NOFT(play n/1 therap*) OR NOFT("case manag*") OR NOFT("social work*") OR NOFT(nurse or nurses or nursing) Or NOFT(reintegrat* or "re-integrat*" or reentry or "re-entry" or resettle* or "re-settle") OR NOFT(Aftercare or "after care") OR NOFT("transitional care"))
S2	(NOFT((psycholog* OR sexual OR physical* OR emotion*) n/2 (harm* or deprivat*)) OR NOFT((psycholog* OR sexual OR physical* OR emotion*) n/2 (abus*))) AND (NOFT(TBI* OR mTBI* OR concuss* OR postconcuss*) OR NOFT((head* or brain* or cerebr* or crani* or skull* or intracran*) n/2 (injur* or trauma* or damag* or wound* or swell* or oedema* or edema* or fracture* or contusion* or pressur*)) OR PUB((head* or brain* or cerebr* or crani* or skull* or intracran*) n/2 (injur* or trauma* or damag* or wound* or swell* or oedema* or edema* or fracture* or contusion* or pressur*)) OR NOFT((brain* or cerebr* or intracerebr* or crani* or intracran* or head* or subdural* or epidural* or extradural* or retinol*) n/1 (haematoma* or hematoma* or hemorrhag* or haemorrhag* or bleed*)) OR NOFT((cogniti* or neurocogniti*) n/2 (impair* or dysfunction* or disorder* or declin*)) OR NOFT(shaken baby)) AND (NOFT(rehab* OR telerehab* OR neurorehab* OR physiatrist* OR physiatry OR Neuropsycholog* OR Nutritionist* or Dietician* OR respite) OR PUB(rehab* OR telerehab* OR neurorehab* OR Neuropsycholog* OR Nutritionist* or Dietician* OR Respite) OR NOFT(occupational n/1 therap*) OR PUB(occupational n/1 therap*) OR NOFT(physical n1 therap*) OR PUB(physical n/1 therap*) OR NOFT(physiotherap* or "physio-therap*") OR PUB(physiotherap* or "physio-therap*") OR NOFT(speech n/2 (therap* or patholog*)) OR PUB(speech n/2 (therap* or patholog*)) OR NOFT(therap* n/1 recreation*) OR PUB(therap* n/1 recreation*) OR NOFT("child life specialist*") OR NOFT(play n/1 therap*) OR NOFT("case manag*") OR NOFT("social work*") OR NOFT(nurse or nurses or nursing) Or NOFT(reintegrat* or "re-integrat*" or reentry or "re-entry" or resettle* or "re-settle") OR NOFT(Aftercare or "after care") OR NOFT("transitional care"))
S3	(NOFT(((violen* or intentional or self-inflict* or other-inflict*) n/6 (head* or brain* or cerebr* or crani* or skull* or intracran*)) n/2 (injur* or trauma* or damag* or wound* or swell* or oedema* or edema* or fracture* or contusion* or pressur*)) OR NOFT(((violen* or intentional or self-inflict* or other-inflict*) n/6 (brain* or cerebr* or intracerebr* or crani* or intracran* or head* or subdural* or epidural* or extradural*)) n/1 (haematoma* or hematoma* or hemorrhag* or haemorrhag* or bleed*)) OR NOFT((violen* or intentional or self-inflict* or other-inflict*) n/6 (tbi*2 or mtbi*2 or concuss* or postconcuss*))) AND (NOFT(rehab* OR telerehab* OR neurorehab* OR physiatrist* OR physiatry OR Neuropsycholog* OR Nutritionist* or Dietician* OR respite) OR PUB(rehab* OR telerehab* OR neurorehab* OR Neuropsycholog* OR Nutritionist* or Dietician*

	OR Respite) OR NOFT(occupational n/1 therap*) OR PUB(occupational n/1 therap*) OR NOFT(physical n1 therap*) OR PUB(physical n/1 therap*) OR NOFT(physiotherap* or "physio-therap*") OR PUB(physiotherap* or "physio-therap*") OR NOFT(speech n/2 (therap* or patholog*)) OR PUB(speech n/2 (therap* or patholog*)) OR NOFT(therap* n/1 recreation*) OR PUB(therap* n/1 recreation*) OR NOFT("child life specialist*") OR NOFT(play n/1 therap*) OR NOFT("case manag*") OR NOFT("social work*") OR NOFT(nurse or nurses or nursing) OR NOFT(reintegrat* or "re-integrat*" or reentry or "re-entry" or resettle* or "re-settle") OR NOFT(Aftercare or "after care") OR NOFT("transitional care"))
S4	S1 OR S2 OR S3

*All English, limited to scholarly journals (excludes dissertations, and conference proceedings)

Grey Literature Search Strategy

Websites of Brain Injury, Violence, and Rehabilitation Organizations (*and CJS and homelessness because of potential overlap) that were searched:

- Abused and Brain Injured Tool-kit
- American Academy of Physical Medicine and Rehabilitation
- Australian Capital Territory Corrective Services
- Brain and Spine Foundation
- Brain Injury Association of America
- Brain Injury Australia (BIA)
- Brain Injury Canada
- Brain Injury Society of Toronto
- Brain Injury New Zealand
- Brain Trauma Foundation
- Brave Program at Sunnybrook Hospital
- British Society of Rehabilitation Medicine
- Canadian Association of Elizabeth Fry
- Canadian Association of Physical Medicine and Rehab
- Canadian Housing First Toolkit
- Canadian Institute for Military and Veteran Health Research
- CDC Foundation for Injury Prevention
- Centre for Crime and Justice Studies
- Cheshire and Merseyside Rehabilitation Network
- Child Brain Injury Trust
- Cochrane Methods Equity Homeless Health Guidelines
- Concussions Ontario
- Connectivity Traumatic Brain Injury Australia
- Criminal Justice Alliance
- Defense and Veterans Brain Injury Center
- Department of Corrections (New Zealand)

- Department of Justice Office on Violence Against Women
- Evidence Exchange Network for Mental Health Addictions
- Federal Bureau of Prisons
- GTA Rehab Network
- Headway : Brain Injury Auckland New Zealand
- House of Commons Canada - Report on Gun Violence
- Human Services and Justice Coordinating Committee
- Injury and Violence Prevention Workgroup
- Irish Penal Reform Trust
- John Howard Society
- John Howard Society of Ontario
- JustSpeak
- L'Allie: Amultimodal approach to remedy behavioural problems and prevent violence in schools
- Law and Justice Foundation of NSW
- LoveYourBrain
- Mental Health Ireland
- Model Systems Knowledge Translation Center
- National Association of State Head Injury
- National Health Care for the Homeless Council
- National Mental Health Consumer and Carer Forum
- Ohio Domestic Violence Network
- Ontario Brain Injury Association
- Queensland Corrective Services
- The Center on Domestic Violence-
  University of Colorado Denver
- The injury and violence prevention network
- The Surge Wraparound: A youth driven plan for gang violence prevention
- Toronto ABI Network
- Toronto Alliance to End Homelessness
- Toronto Mental Health and Addictions Supportive Housing Network
- U.S. Department of Justice
- United Kingdom Acquired Brain Injury Forum
- Veterans & Families Research Hub
- Violence Impact Program
- Women with Disabilities Victoria
- Women's National Housing and
- Homelessness Network

The following keywords were used to search for relevant grey literature reports:

- Concept A: violence, interpersonal violence, assault, intentional injury, family violence, and community
- Concept B: rehabilitation, neuropsychologists, nurse, nutritionist, occupational therapist, physiatrist, physician, physiotherapist, psychologist, psychometrist, social worker, speech language pathologist,

therapeutic recreationist
- Concept C: traumatic brain injury, brain injury; cognitive impairment, disability, trauma

**Concept**	**Keyword**
**A. Violence**	interpersonal violence or assault or intentional injury or family violence or community violence
**B. Rehabilitation**	Neuropsychologists or nurse or nutritionist, or occupational therapist or physiatrist or physician, physiotherapist or psychologist or psychometrist or social worker or speech language pathologist or therapeutic recreationist
**C. Traumatic Brain Injury**	brain injury or concussion or brain trauma or cognitive impairment or head trauma or TBI

Final Search Looking For:

A + B + C
