# Supplementary material for: Rehabilitation and violence-related traumatic brain injury: A scoping review
Source: PLoS One. 2024 Nov 14;19(11):e0310803. doi: 10.1371/journal.pone.0310803 (PMC11563373; doi:10.1371/journal.pone.0310803)
Supplement: S2 File — (PDF) [file pone.0310803.s003.pdf]

Supplementary File 2

Charting Table

Study (Author, Year, Country)	Study Design & Objective	Study Sample	TBI Status	Violence Status	Rehabilitation Intervention, Team, Outcome	TBI-Specific Barriers, Facilitators, and Gaps
**Ackerman & Banks, 2003**<br><br>**United States**	Case study<br><br>Focuses on assessment and treatment for the neuropsychological consequences of head injury sustained through intimate partner violence	N = 3<br><br>Age range: 46 – 48 years<br><br>Sex/Gender [not specified]*: All women<br><br>Ethnicity: African American, European American<br><br>Other: Stressors related to unemployment and difficult transitions with family	N = 3(100 %)<br><br>Definition: 2 Clients with TBI sustained through violence (1 through Motor vehicle accident, 2 through physical abuse)<br><br>Method of identification: NR<br><br>Severity: NR<br><br>Time since injury: NR	N = 2(66 %)<br><br>Physical abuse	Intervention: Psychotherapy, bio feedback<br><br>Team: Psychotherapist<br><br>Outcome: Positive self-reported outcomes related to depression, relaxation, and neuropsychological function	Barrier: Fear of ex-husband leading to difficulty receiving therapy<br><br>Facilitator: The use of neuropsychological tests and neuropsychological-informed psychotherapy to guide treatments for mild TBI<br><br>Gap: Important to identify if clients sustained head injuries to ensure neuropsychological problems are adequately addressed
**Ajir, F., & Tibbets, J., 1981**<br><br>**United States**	Case study<br><br>To report the case of an 18-year-old man who sustained a minor head and neck injury during a fist fight and developed a left hemiparesis resulting from thrombosis of the supraclinoid portion of the right internal carotid artery	N = 1<br><br>Age: 18 years<br><br>Sex/Gender [not specified]*: Male<br><br>Race/ethnicity: NR<br><br>Other: N/A	N = 1(100 %)<br><br>Definition: Minor head and neck injury during fist fight leading to left hemiparesis<br><br>Method of identification: CT scan<br><br>Time since injury: 48 hours<br><br>Severity: NR	N = 1(100 %)<br><br>Hit on right temple by a fist during a fight and was held around the neck by a forearm for a short time	Intervention: Aggressive physical therapy<br><br>Team: Physical therapist<br><br>Outcome: Able to walk without assistance after two months	Barrier: NR<br><br>Facilitator: Considering vascular injuries for patients with head injuries and pursuing neurological evaluation with angiography for unexplained neurological deficits<br><br>Gap: NR
**Alaca R, Yilmaz B, Gunduz S., 2002**<br><br>**Turkey**	Case study<br><br>To report a case of onset of diabetes insipidus during acute rehabilitation of a 20-yr-old patient with a	N = 1<br><br>Age: 20<br><br>Sex/Gender [not specified]*: Male<br><br>Race/ethnicity: NR	N = 1 (100 %)<br><br>Definition: Penetrating gunshot injury through right temporal region<br><br>Method of identification: NR	N = 1 (100 %)<br><br>Penetrating gunshot injury through right temporal region	Intervention: Acute rehabilitation unit<br><br>Team: NR<br><br>Outcome: NR	Barrier: Late initiation of treatment can be accepted as a factor for poor prognosis<br><br>Facilitator: NR<br><br>Gap: NR

Supplementary File 2

Charting Table

Study (Author, Year, Country)	Study Design & Objective	Study Sample	TBI Status	Violence Status	Rehabilitation Intervention, Team, Outcome	TBI-Specific Barriers, Facilitators, and Gaps
	traumatic brain injury caused by a gunshot wound	Other: N/A	Time since injury: 12 days<br><br>Severity: NR			
**Aras et al., 2004**<br><br>**Turkey**	Quantitative descriptive<br><br>To describe the demographic characteristics and the nature of the functional recovery in a group of Turkish survivors of TBI who were referred for inpatient rehabilitation and identify variables correlated with discharge functional status as measured by the Functional Independence Measure	N = 40<br><br>Age (mean ± SD): 28 ± 9.8 years<br><br>Sex/Gender [not specified]*:<br>32 (69.6 %) male and eight (17.4 %) female<br><br>Race/ethnicity:<br>Turkish<br><br>Other: N/A	N = 40(100 %)<br><br>Definition: Brain injury is defined as acquired and non-progressive; traumatic brain injury (TBI) is the most common form<br><br>Method of identification: NR<br><br>Time since injury: NR<br><br>Severity: NR	N = 9 (22.5 %)<br><br>Violence	Intervention: Inpatient rehabilitation encompassing daily physiotherapy, occupational therapy, speech therapy, psychotherapy, nutrition and dietary services improvement<br><br>Team: Physiotherapist, occupational therapist, speech therapist, psychotherapist, nutrition and dietary services (professional not specified)<br><br>Outcome: Significant improvements the Disability Rating Scale, Functional Independence Measure, and the Rancho Los Amigos Levels of Cognitive Functioning Scale from the time of admission to the time of discharge	Barrier: Longer durations of coma and PTA have also been associated with poorer neurological and functional outcome. In this TBI population, the delay in admission to rehabilitation seems to decrease the functional gains and increase the length of rehabilitation stay.<br><br>Facilitator: NR<br><br>Gap: Difficulty differentiating the contribution of spontaneous recovery from rehabilitation
**Bogner et al., 2001**<br><br>**United States**	Longitudinal study<br><br>To determine the relative contributions of substance abuse history and violent etiology to the prediction of outcomes for individuals who sustained a TBI	N=351 (100 %)<br><br>Age (mean): Violent etiology = 31.45, Non-violent = 33.67<br><br>Gender:<br>Violent etiology:<br>Men (96 %)<br>Women (4 %)<br><br>Non-violent etiology:	N=351 (100 %)<br><br>Definition: NR<br><br>Method of identification: NR<br><br>Time since injury: NR<br><br>Severity:	N=53 (15 %)<br><br>Violence etiology was defined as an injury resulting from assault or a gunshot wound	Intervention: Specialized TBI acute rehabilitation unit<br><br>Team: NR<br><br>Outcome: A trend suggested a relationship with violent etiology, with those with an injury of violent etiology being less competent in the home 1 year after discharge from rehabilitation	Barrier: NR<br><br>Facilitator: NR<br><br>Gap: Generalizations to the population of persons with TBI who receive rehabilitation should be made with caution

Supplementary File 2

Charting Table

Study (Author, Year, Country)	Study Design & Objective	Study Sample	TBI Status	Violence Status	Rehabilitation Intervention, Team, Outcome	TBI-Specific Barriers, Facilitators, and Gaps
	requiring inpatient rehabilitation.	Men (76 %)<br>Women (24 %)<br><br><u>Race:</u><br>Violent etiology:<br>White (68 %)<br>Other (32 %)<br><br>Non-violent etiology:<br>White (96 %)<br>Other (4 %)	65 % severe, 13 % moderate, and 22 % mild TBI			
**Brain Injury Society of Toronto, 2019**<br><br>**Canada**	Mixed methods<br><br>To identify how the housing first model supports individuals in chronic homelessness and/or are experiencing a brain injury	N = 7<br><br><u>Age</u> range: 42 - 66<br><br><u>Sex:</u> 5 males, 2 females<br><br><u>Ethnicity:</u> NR<br><br><u>Other:</u><br>• 94 % relied on social assistance/financial benefit<br>• 19 entered the program insecurely housed<br>• 69 % experienced issues with mental health<br>• 97 % were unemployed at the beginning and throughout the duration of support.<br>• 31 % of were dealing with substance abuse	N = 32 Brain injured individuals, however only 7 specified qualitative cases<br><br><u>Definition</u>: A brain injury is damage to the brain that can be acquired (ABI) after birth due to factors such as: stroke, brain tumour, suffocation, substance abuse or poisoning. A brain injury can also be traumatic in nature (TBI) and may be caused by incidents such as a car accident, fall, assault, domestic abuse or sports injury.<br><br><u>Method of identification</u>: NR<br><br><u>Time since injury</u>: NR<br><br><u>Severity</u>: NR	Only specified cases of assault n = 2 (Edward and Richard).<br><br>IPV	<u>Intervention</u>: Housing First – Housing is provided first along with supports<br><br><u>Team</u>: Doctors, case managers<br><br><u>Outcome:</u><br>• 34 % of participants were supported in locating a family physician<br>• Relationships were formed with 14 landlords throughout the program<br>• 64 % of Program Participants were successful in securing housing<br>• Outcome for assault-related cases:<br>- Obtained secure housing with access to nutritious food and continues to be supported via long-term case management after a few months<br>- Accepted an offer for subsidized housing after 5 months and connected to long-term case	<u>Barrier:</u><br>• Lack of familiarity with the city causing difficulty in setting appointments<br>• Substance use and isolation when distressed<br>• No access to a cellphone<br>• Illiteracy hindering computer and email use and creating barriers to communication and scheduling<br><br><u>Facilitator:</u> Securing suitable housing and financial security<br><br><u>Gap:</u> NR

Supplementary File 2

Charting Table

Study (Author, Year, Country)	Study Design & Objective	Study Sample	TBI Status	Violence Status	Rehabilitation Intervention, Team, Outcome	TBI-Specific Barriers, Facilitators, and Gaps
					management and a program to manage substance use	
**Brain Injury Society of Toronto, 2020**<br><br>**Canada**	Mixed methods<br><br>To outline the correlation between IPV and brain injury	N = 33<br><br>Age range: 30 - 64 years<br><br>Sex: 75 % females, 25 % males<br><br>Ethnicity: NR<br><br>Other:<br>• 39 % of program users suffered from mental health issues<br>• 32 % of the individuals served were living below the poverty line	61 % formal TBI diagnosis 39 % clear signs of brain injury and history of brain injuries<br><br>Definition: NR<br><br>Method of identification: 61 % formal brain injury diagnosis; NR for 39 % but noted that they had clear signs of brain injury and a history of brain injuries<br><br>Time since injury: NR<br><br>Severity: NR	100 % - 33 referred to the Violence Impact Program<br><br>IPV	Intervention: Violence impact program<br>• Income support<br>• Referral to community resources<br>• Connecting with physicians<br>• Securing suitable housing<br>• Education on trauma-informed care<br>• Food security<br><br>Team: Violence impact coordinator, specialist, family doctor<br><br>Outcome: NR	Barrier: Lack of training regarding dealing with the consequences of TBI among agencies that work for survivors of IPV; as a result, most services focus on treating psychological well-being and neglecting issues associated with TBI (e.g., cognitive processing, memory, and focus/attention)<br><br>Facilitator: NR<br><br>Gap: NR
**Brown et al., 2019**<br><br>**United States**	Quantitative<br><br>The current study explores the use of Neurofeedback to treat IPV survivors who experienced head injury and, as such, probable TBI	N = 32<br><br>Age (mean): 46.9<br><br>Gender: 31 females, 1 male<br><br>Ethnicity: White (63 %), Hispanic (19 %), Black or African American (13 %), and Asian (5 %)<br><br>Other: N/A	N = 32 (100 %)<br><br>Definition: TBI is an injury to the brain typically caused by an acute injury to the head, neck, or face, such as a blunt force trauma, contusion, or strangulation<br><br>Method of identification: NR<br><br>Time since injury: NR<br><br>Severity: NR	N=32 (100 %) all participants sustained TBI through IPV	Intervention: Neurofeedback<br><br>Team: Clinicians (not specified)<br><br>Outcome: NR	Barrier: Lack of training regarding dealing with the consequences of TBI among agencies that work for survivors of IPV; as a result, most services focus on treating psychological well-being and neglecting issues associated with TBI (e.g., cognitive processing, memory, and focus/attention)<br><br>Facilitator: NR<br><br>Gap: NR

Supplementary File 2

Charting Table

Study (Author, Year, Country)	Study Design & Objective	Study Sample	TBI Status	Violence Status	Rehabilitation Intervention, Team, Outcome	TBI-Specific Barriers, Facilitators, and Gaps
**Burnett et al. 2003**<br><br>**United States**	Retrospective analysis<br><br>To compare demographics, injury characteristics, therapy service and intensity, and outcome in minority versus nonminority patients with traumatic brain injury	N = 1020<br><br>Age (mean): 35.4<br><br>Gender: 502 females, 518 males<br><br>Ethnicity: nonminority 168, minority 852<br><br>Other: N/A	N=2020 (100 %) experienced TBI<br><br>Definition: NR<br><br>Method of identification: Traumatic brain injury (TBI) has been investigated by using the Traumatic Brain Injury Model Systems (TBIMS) database.<br><br>Time since injury: NR<br><br>Severity: NR	Only referred to as 'acts of violence'<br><br>Minorities (n=852), 33.4 % with act of violence etiology.<br><br>Non-Minority (n=1168), 11.4 % with act of violence etiology.	Intervention: Inpatient rehabilitation<br><br>Team: Nursing, occupational therapy, physiatrist, related medical services, physical therapy, psychologic and neurologic assessment, speech language pathology, recreation therapy, and social services<br><br>Outcome: No significant differences in outcome variables, including rehabilitation charges, discharge disposition, FIM scores, FIM score changes, and post injury employment status.	Barrier: NR<br><br>Facilitator: NR<br><br>Gap: Inclusion of detailed socioeconomic information, as well as consideration of cultural variables, may help explain outcome differences and simultaneously increase provider awareness
**Callahan, C. D.; Hagglund, K. J., 1995**<br><br>**United States**	Case report<br><br>Describes the case of a 20-year-old man who sustained a gunshot wound to the forehead, resulting in traumatic brain injury and C2 ventilator-dependent quadriplegia	N = 1<br><br>Age: 20<br><br>Gender: Male<br><br>Race/ethnicity: NR<br><br>Other: Brief inpatient psychiatric hospitalization several years before his injury, on the death of a parent after a long illness.	Definition: A gunshot wound to the forehead, resulting in traumatic brain injury<br><br>Method of identification: NR<br><br>Time since injury: NR<br><br>Severity: NR	Gunshot wound resulting in TBI	Intervention: Acute inpatient rehabilitation<br><br>Team: Psychiatrist, rehabilitation psychologist<br><br>Outcome: NR	Barrier: NR<br><br>Facilitator: Medical ethics review to discuss patient's decision to refuse care and to address the issue of competency<br><br>Gap: NR
**Chowdhury, S.; Leenen, L. P. H., 2021**<br><br>**Kingdom of Saudi Arabia**	Retrospective observational<br><br>To compare the outcomes regarding the discharge	N = 219<br><br>Age: 28.2<br><br>Sex: 195 males (89 %)	N = 2,021 (59.1 %) patients sustained TBI, only n = 219 selected for study<br><br>Definition: NR	n=5 (2.3 %)<br><br>Assault	Intervention: Acute in-hospital intensive rehabilitation program for a selected group of severely injured trauma patients who receive at least two to three	Barrier: Long waiting list leading to delay in transfers of patients and increasing hospital stay and cost; long waits for rehabilitation have a negative impact on the

Supplementary File 2

Charting Table

Study (Author, Year, Country)	Study Design & Objective	Study Sample	TBI Status	Violence Status	Rehabilitation Intervention, Team, Outcome	TBI-Specific Barriers, Facilitators, and Gaps
	destination and length of hospital stay of selected TBI patients before and after launching an acute intensive trauma rehabilitation (AITR) program at King Saud Medical City	Ethnicity: Saudi Arabian<br><br>Other: N/A	Method of identification: For these selected patients, the data of patient demographics, mechanism of injuries, baseline admission characteristics (on presentation to ED), length of stay, and discharge destination in terms of home or rehabilitation center were extracted from the trauma registry.<br><br>Time since injury: NR<br><br>Severity: NR		sessions of different therapies, including physiotherapy, speech-language therapy, occupational therapy and Botox therapy for 3 to 4 hours each day with breaks in between and five days a week as decided by the physiatrist.<br><br>Team: Physiatrists, physiotherapy, occupational therapy, speech-language pathology, prosthesis and orthosis, and the social work department<br><br>Outcome:<br>• Reduced referrals to another rehabilitation or long-term care facility<br>• Reduced length of stay in the hospital	functional and cognitive recovery of severely injured patients<br><br>Facilitator: NR<br><br>Gap: Identifying factors that contribute to the prediction of discharge disposition is crucial for efficient resource utilization and reducing cost. Several factors may influence discharge location after hospitalization
**Conway et al., 1999**<br><br>**United States**	Case study<br><br>The paper describes ReMed, a community-based brain injury program, specializes in using a behavior analytic approach to address unwanted behaviors while understanding and developing strategies to further recovery from the brain injury.	N = 1<br><br>Age: 36<br><br>Sex/Gender [not specified]*: Male<br><br>Race/ethnicity: NR<br><br>Other: NR	N = 1 (100 %)<br><br>Definition: A traumatic assault to the brain often leaves significant residual effects, including severe behavioral dyscontrol. The person may demonstrate unwanted behaviors, such as physical aggression or verbal abuse, and lack motivation or the skills to engage in desirable behavior.<br><br>Method of identification: NR	N = 1 (100 %)<br><br>Assault	Intervention: Neurobehavioral short-term rehabilitation system at ReMed - a community-based brain injury program that uses a behavior analytic approach to address unwanted behaviours while understanding and developing strategies to further recovery from brain injury<br><br>Team: NR<br><br>Outcome:<br>• Decreased frequency of negative behaviours	Barrier: Use of extinction to address unwanted behaviours was counterintuitive and involved certain risks (e.g., increases in both the intensity and frequency of targeted behaviors and the emergence of other maladaptive behaviors to gain attention).<br><br>Facilitator:<br>• Informing the staff and family of the likely increase in behaviors resulting from extinction procedures<br>• Assessing a program's ability to tolerate increased frequency and intensity of targeted behaviors

Supplementary File 2

Charting Table

Study (Author, Year, Country)	Study Design & Objective	Study Sample	TBI Status	Violence Status	Rehabilitation Intervention, Team, Outcome	TBI-Specific Barriers, Facilitators, and Gaps
			Severity: NR<br><br>Time since injury: NR		• Requires less intervention and staff support<br>• Interviewed for a vocational opportunity	before beginning any extinction procedure.<br>• Identifying target behaviors and reinforcers in the behavioral plan to manage risks while shaping appropriate behaviors<br><br>Gap: NR
**de Guise et al., 2017**<br><br>**Canada**	Quantitative<br><br>To identify factors that would predict short-term neuropsychological outcome in patients with TBI hospitalized in an acute rehabilitation setting	N = 348<br><br>Age (mean): 40.19 of entire sample, mean age of sample affected by violence<br><br>Sex/Gender [not specified]*: NR<br><br>Race/ethnicity: NR<br><br>Other: Mean level of education was 11.51 years	N=348 (100 %)<br><br>Definition: TBI can cause not only physical symptoms, but also cognitive, emotional and behavioural malfunction or impairment. Indeed, cognitive and behavioural dysfunctions are usually observed after a traumatic brain injury (TBI).<br><br>Method of identification: NR<br><br>Severity: NR<br><br>Time since injury: NR	N=70 (20.1 %)<br><br>Assault	Intervention: TBI programme<br><br>Team: Rehabilitation nurse specialist, clinical neuropsychologist<br><br>Outcome: NR	Barrier: NR<br><br>Facilitator: NR<br><br>Gap:<br>• Exclusion of patients with pre-morbid history of alcohol and drug abuse and those with various impairments created a selection bias<br>• Generalization to the broader population of patients with mild TBI should be done with caution<br>• Limited number of tests used; does not give a complete neuropsychological profile
**Dhamapurkar et al., 2016**<br><br>**Canada**	Case study (Retrospective design)<br><br>To suggest that continuing improvement is possible in a survivor of	N = 1<br><br>Age: 29<br><br>Sex/Gender [not specified]*: Man<br><br>Race/ethnicity: NR	N = 1 (100 %)<br><br>Definition: Traumatic brain injury (TBI) due to an assault in which a baseball bat was used, which led to immediate coma. He suffered multiple facial fractures, left orbital	N = 1 (100 %)<br><br>Assault using a baseball bat	Intervention:<br>• Rehabilitation from a sub-acute unit after a month of acute hospitalization in intensive care<br>• Transferred to a specialized brain rehabilitation unit and assigned to multidisciplinary team for an early intensive	Barrier: NR<br><br>Facilitator:<br>• Intensive therapy and regular assessments - received daily occupational therapy and physiotherapy. He received neuro functional re-organization therapy and speech and language therapy

Supplementary File 2

Charting Table

Study (Author, Year, Country)	Study Design & Objective	Study Sample	TBI Status	Violence Status	Rehabilitation Intervention, Team, Outcome	TBI-Specific Barriers, Facilitators, and Gaps
	catastrophic brain injury after being in a prolonged state of disordered consciousness	Other:<br>Support from family	fractures and temporal-parietal skull fractures<br><br>Method of identification: NR<br><br>Severity: NR<br><br>Time since injury: NR		rehabilitation program at 4 months post-injury<br><br>Team: Art therapist, physiotherapist and occupational therapist, neuro functional re-organization therapy and speech and language therapy<br><br>Outcome: Despite a devastating brain injury and a long period in a state of disordered consciousness, patient regained full consciousness, is speaking normally, has a reasonable memory, is learning to walk again, is playing the guitar and is functioning in the community with minimal support.	twice a week, music and art therapy once or twice weekly; he was regularly assessed; and he was not allowed to lie in bed doing nothing and this may be one of the main factors enabling him to improve.<br>• Cranioplasty 10 months post craniectomy<br>• Support from family<br><br>Gap:<br>Future research is required to determine whether the gains seen in patient can be generalized to patients with other aetiologies using a similar treatment regime.
**Donelly et al., 2019**<br><br>**United States**	Qualitative<br><br>To explore participants ‘experiences in a group-based yoga with psychoeducation intervention designed to facilitate community integration for people with traumatic brain injury and their caregivers	N = 16<br><br>Age range:<br>18 to 34 years (n = 5)<br>35 to 54 years (n = 6)<br>55 years or older (n = 5)<br><br>Sex:<br>Female (n = 14)<br>Male (n = 2)<br><br>Race/ethnicity[not specified]**:<br>White (n = 15)<br>Asian (n = 1)<br>Non-Hispanic (ethnicity; n = 16)	n = 13 (81.25 %)<br><br>Definition: NR<br><br>Method of identification: NR<br><br>Severity: TBI ranges in severity from mild to severe and can lead to a multitude of physical, cognitive, behavioral, and psychosocial deficits that compromise quality of life.<br>N =16 Number of TBIs<br>1 - 11<br>2 - 1	n = 2 (7.69 %)<br><br>Assault	Intervention: LoveYourBrain Yoga, a 6-session, manualized, group-based yoga intervention that incorporates breathing exercises, yoga, meditation, and psychoeducation<br><br>Team: NR<br><br>Outcome:<br>• Participants reported improvements in strength, balance, flexibility, and attention control, and a greater sense of belonging, community connection, and	Barrier: Scheduling<br><br>Facilitator: NR<br><br>Gap: NR

Supplementary File 2

Charting Table

Study (Author, Year, Country)	Study Design & Objective	Study Sample	TBI Status	Violence Status	Rehabilitation Intervention, Team, Outcome	TBI-Specific Barriers, Facilitators, and Gaps
		Other:<br>• Employment (Currently employed)<br>Yes (n = 11)<br>No (n = 5)<br><br>• Education<br>High school graduate or less (n = 0)<br>College or some college (n = 8)<br>More than a Bachelor's (n = 8)	3 - 1<br><br>Time since injury: Mean years since TBI (SD) 7 (6.7)		ability to move forward with their lives.<br>• Participants reported ongoing use of tools (e.g., breathing exercises) to cope with negative emotions and stress.<br>• About half of participants sustained relationships built during LoveYourBrain Yoga and felt more capable of accessing other activities in their community.	
**Donnelly et al., 2021**<br><br>**United States**	Retrospective study<br><br>To conduct a mixed methods, pre-post, retrospective study on the feasibility, acceptability, and effectiveness of the LoveYourBrain Yoga program	N = 1563<br><br>Age range:<br>15–34 213 (30.3 %)<br>35–54 326 (46.3 %)<br>55–70 165 (23.4 %)<br>Missing 1<br><br>Sex:<br>Male 173 (24.6 %)<br>Female 530 (75.4 %)<br>Missing 2<br><br>Race/ethnicity: NR	N = 705<br><br>Definition: NR<br><br>Method of identification: NR<br><br>Severity:<br>Mild 326 (46.3 %)<br>Moderate 181 (25.7 %)<br>Severe 197 (28.0 %)<br><br>Time since injury: NR	N = 29 (4.3 %)<br><br>Assault, struck by or against	Intervention: 6-week yoga and psychoeducation<br><br>Team: NR<br><br>Outcome:<br>• No improvement was found in Emotional and Behavioral Dysregulation, however, content analysis revealed better ability to regulate anxiety, anger, stress, and impulsivity.<br>• Caregivers perceived improvements in physical and psychological health	Barrier:<br>• Scheduled during work hours, peak traffic, or too early in the morning<br>• Location requires a far commute<br><br>Facilitator: NR<br><br>Gap: Need to utilize more robust study designs to more reliably assess the extent to which outcome changes can be attributed to the intervention
**Eismann et al., 2020**<br><br>**United States**	Quantitative<br><br>To assess early developmental, behavioral, and quality of life outcomes following	N=107<br><br>Age: Under 12 months old<br><br>Gender:<br>Attended postinjury clinic: Male 24	N=107 (100 % abusive head trauma history)<br><br>Definition: Pediatric abusive head trauma is an injury to the head of a young child that results	N=107 (100 % abusive head trauma history)<br><br>Abusive head trauma	Intervention: Early therapeutic intervention, physical or occupational therapy<br><br>Team:<br>• Child abuse pediatrician<br>• Trained nurse practitioner	Barrier: NR<br><br>Facilitator: Social worker performs psychosocial assessment and referrals

Supplementary File 2

Charting Table

Study (Author, Year, Country)	Study Design & Objective	Study Sample	TBI Status	Violence Status	Rehabilitation Intervention, Team, Outcome	TBI-Specific Barriers, Facilitators, and Gaps
	infant abusive head trauma and evaluate injury severity and early therapeutic intervention as potential predictors	Female 11<br>Completed Mullen Scale:<br>Male 19<br>Female 6<br>n=116<br>Male: attended post injury clinic, no - 24 (69 %), yes 44 (61 %)<br><br>Female: attended post injury clinic, no - 11 (31 %), yes - 28 (39 %)<br><br><u>Race:</u><br>White & attended post injury clinic<br>• No - 24<br>•Yes - 49<br><br>Black & attended post injury clinic:<br>• No - 7<br>• Yes - 14<br><br>More than one race: attended post injury clinic<br>• No - 0<br>• Yes - 3<br><br>Ethnicity: nonhispanic/Latino<br>• No - 28<br>• Yes - 63<br><br>Hispanic/Latino<br>• No - 7<br>• Yes - 7<br><br><u>Other:</u> N/A	from inflicted blunt impact and/or violent shaking<br><br><u>Method of identification</u>: NR<br><br><u>Severity</u>: NR - only mentioned mild, moderate, and severe TBI<br><br><u>Time since injury</u>: NR		• Physical therapist<br>• Occupational therapist<br>• Speech therapist<br>• Special education<br><br><u>Outcome:</u> NR	<u>Gap:</u> Additional research with larger samples is encouraged to better understand the effectiveness of different types of rehabilitative services for patients following abusive head trauma.

Supplementary File 2

Charting Table

Study (Author, Year, Country)	Study Design & Objective	Study Sample	TBI Status	Violence Status	Rehabilitation Intervention, Team, Outcome	TBI-Specific Barriers, Facilitators, and Gaps
**Esselman et al., 2004**<br><br>**United States**	Cohort study<br><br>To examine injury characteristics, demographics, and discharge disposition after traumatic brain injury of violent or nonviolent cause	N = 1807<br>Violence (n =286)<br><br>Age (mean): violence = 34.8<br><br>Sex:<br>Men in violence group – 90.6 %<br><br>Race (%)<br>White – 55.9 %<br>African American – 26.6 %<br>Asian – 4.9 %<br>Hispanic – 8.0 %<br>Native American – 4.5 %<br><br>Other:<br>• Medicaid was the funding source for more of the violently injured than for the non-violently injured<br>• Higher rate of self-pay for violently injured and difference in rates of self-pay between groups reflects shorter length of stay in the violence-related group.	N=1807 (100 % sustained brain injuries)<br><br>Definition: NR<br><br>Method of identification: A Head AIS of 2 or more includes skull fractures, documented LOC, neurologic deficits, or head computed tomography (CT) scan abnormalities, but it eliminates scalp lacerations without documented brain injury N=1807 (100 % sustained brain injuries)<br><br>Severity: NR<br><br>Time since injury: NR	N=286 (15.8 %)<br><br>"A Head AIS of 2 or more includes skull fractures, documented LOC, neurologic deficits, or head computed tomography (CT) scan abnormalities, but it eliminates scalp lacerations without documented brain injury."<br><br>Specific mechanism of injury NR	Intervention: Inpatient rehabilitation<br><br>Team: Healthcare practitioners (not specified)<br><br>Outcome: NR	Barrier:<br>• Some veterans may not have any friends or family that can support them or that they trust to undergo the intervention with<br>• Some patients who have Medicaid were more likely to get discharged to skilled nursing facilities (SNF) instead of inpatient rehab despite having a violence related injury<br><br>Facilitator: NR<br><br>Gap:<br>Included only patients who were admitted to inpatient rehabilitation and did not include patients who were discharged from acute care to home or to nursing facilities
**Fraser et al., 2019**<br><br>**Australia**	Longitudinal study<br><br>To examine the association of injury severity measured by PTA duration, age, and cognitive	N = 109<br><br>Age (mean ± SD): 44.62 ± 16.72<br>Range 20–85<br><br>Gender (n%):	n = 109 (100 %)<br><br>Definition:<br><br>Method of identification: Mean PTA duration of 28.17 days (SD = 48.95)	N = 2 (2 %)<br><br>Assault	Intervention:<br>• 3–5 hours of daily occupational therapy, physiotherapy, and speech therapy, neuropsychological assessment, and social work services	Barrier: NR<br><br>Facilitator: NR<br><br>Gap: NR

Supplementary File 2

Charting Table

Study (Author, Year, Country)	Study Design & Objective	Study Sample	TBI Status	Violence Status	Rehabilitation Intervention, Team, Outcome	TBI-Specific Barriers, Facilitators, and Gaps
	reserve (indicated by pre-morbid IQ) with recovery of cognitive functions between two and five years after TBI	Female 32(29.4 %)<br>Male 77(70.6 %)<br><br>Race/ethnicity: NR<br><br>Other: N/A	and a mean GCS score of 9.47 (SD = 4.33)<br><br>Severity: The majority of participants (41 %) had sustained a severe TBI, based on a mean PTA duration<br><br>Time since injury: NR		• Followed generally by outpatient or community-based rehabilitation, with continuing therapy as needed<br><br>Team: Occupational therapy, physiotherapy, and speech therapy, neuropsychological assessment, and social work services<br><br>Outcome: NR	
**Gutman et al., 2004**<br><br>**United States**	Quasi-experimental<br><br>To assess the effectiveness and client acceptability of an intervention for women experiencing domestic violence and/or homelessness, whose cognitive impairments may have contributed to their inability to leave an abusive situation	Age:<br>Mean age: 44 years old– the youngest participant was 23; the oldest, 58. 23 % (6) of the participants were in their 20s; 8 % (2) were in their 30s; 27 % (7) were in their 40s; and 42 % (11) were in their 50s.<br><br>Gender: Women (n = 26)<br><br>Race/ethnicity [not specified]**:<br>42 % (11) African-American; 35 % (9) Caucasian; 15 % (4) Latino; and 8 % (2) Indian.	N=3 (19 %)<br><br>Definition: NR<br><br>Method of identification: NR<br><br>Severity: Nineteen percent (3) of the women who reported domestic abuse had sustained traumatic brain injury (TBI) severe enough to both receive medical treatment and a diagnosis of TBI. Such non-organic cognitive damage took the form of poor concentration and judgment, and decreased problem solving, planning, and decision-making–all cognitive deficits that are characteristic of mild brain damage. It is suggested that such cognitive impairment	N=3 (19 %)<br><br>Solely referred to as domestic abuse/violence	Intervention:<br>Occupational therapy intervention that primarily addressed how the women's cognitive deficits could be remediated (or compensated for) in order to help them rebuild lives free of abuse. The intervention encompassed:<br>• Safety planning<br>• Drug and alcohol awareness<br>• Safe sex practices<br>• Assertiveness and advocacy skill training<br>• Anger management<br>• Stress management<br>• Boundary establishment and limit setting<br>• Vocational and educational skill training<br>• Money management<br>• Housing application<br>• Leisure exploration	Barrier:<br>• Many of the women who were homeless also experienced past abuse but never disclosed this and failed to receive appropriate services. Such women may have been inappropriately placed in co-ed therapy groups and shared a shelter that also housed homeless men.<br>• Women would also get diagnosed with PTSD or other conditions that lead to different treatment.<br><br>Facilitator: NR<br><br>Gap:<br>• Difficult to generalize the results to other populations who experience domestic violence<br>• Epidemiological studies designed to determine the percentage of women sustaining brain injury as a result of

Supplementary File 2

Charting Table

Study (Author, Year, Country)	Study Design & Objective	Study Sample	TBI Status	Violence Status	Rehabilitation Intervention, Team, Outcome	TBI-Specific Barriers, Facilitators, and Gaps
			may have resulted from mild brain damage sustained through domestic abuse occurring over time.<br><br>Time since injury: NR		• Hygiene, medication routine, and nutrition<br><br>Team: Social workers, case managers, occupational therapists, physicians, and nurses. Clinical services include individual and family counseling, recreational groups, life skill training, and medical services<br><br>Outcome: 81 % (21) of the 26 participants attained T scores above 50, indicating that they achieved their most favorable outcome (according to their GAS scores). Of the 16 participants who had been admitted into the domestic violence program, 87.5 % (14) attained T scores greater than 50. Seventy percent (7) of the 10 participants admitted into the homeless program attained T scores above 50.	domestic violence is sorely needed.
**Hart et al., 2017**<br><br>**United States**	RCT<br><br>To test efficacy of 8-session, 1:1 treatment, anger self-management training (ASMT), for chronic moderate to severe TBI	N = 90<br><br>Age:<br>Mean age<br>• PRE = 36.2<br>• ASMT = 30.4<br><br>Sex (%)<br>• PRE – males (80 %)<br>• ASMT – males (82 %)<br><br>Race (n%)<br>PRE	N=90 (100 %) sustained TBI<br><br>Definition: NR<br><br>Method of identification:<br>NR; TBI-related variables such as mechanism of injury and severity were ascertained using a structured interview that has been used in other studies of chronic TBI. All	N=12 (29 %)<br><br>Intentional injury	Intervention:<br>ASMT protocol emphasizes the teaching of behavioral skills (self-monitoring, problem-solving) in addition to focused education about anger and its links to TBI.<br><br>PRE(personal readjustment and education) protocol provides education about the effects of TBI on personal characteristics, relationships,	Barrier: NR<br><br>Facilitator: NR<br><br>Gap:<br>All participants resided in the community and were excluded for serious limitations in communication. Thus, it cannot be assumed that the concepts and techniques taught in the ASMT would be accessible to

Supplementary File 2

Charting Table

Study (Author, Year, Country)	Study Design & Objective	Study Sample	TBI Status	Violence Status	Rehabilitation Intervention, Team, Outcome	TBI-Specific Barriers, Facilitators, and Gaps
		• White – 22(73 %)<br>• Black – 8(27 %)<br>• Hispanic/other – 0(0 %)<br><br>ASMT<br>• White - 40(67 %)<br>• Black - 13(22 %)<br>• Hispanic/other - 7(12 %)<br><br>Other: N/A	participants' TBI severity was confirmed for study inclusion using prospective medical records<br><br>Severity: NR<br><br>Time since injury: NR		and community roles. It proscribes advice or guidance from the therapist in favor of empathetic listening, reflection, and encouragement to pursue one's own posttraumatic readjustment.<br><br>Team: Psychologist, study therapists (not specified)<br><br>Outcome: Primary Outcome: Response to treatment defined as 1 or more standard deviation change in self-reported anger. Secondary Outcomes: SO-rated anger, emotional and behavioral status, satisfaction with life, timing of treatment response, participant and SO-rated global change, and treatment satisfaction	those with very severe cognitive or communication deficits.<br><br>Results would not generalize to people with TBI who deny anger problems that are apparent to others, as all participants had to self-report problems with anger in order to enter the trial.
**Hart et al.,2012**<br><br>**United States**	Pilot study<br><br>To examine the feasibility and gather preliminary data on the efficacy of a fully manualized, 8-session, psychoeducational treatment for irritability and anger after TBI, called anger self-	N = 10<br><br>Age range: 23 - 59<br><br>Sex:<br>Males (n = 8)<br>Females (n = 2)<br><br>Race:<br>White – 6 (60 %)<br>African American – 4(40 %)<br><br>Other: N/A	N=10 (100 %) sustained TBI<br><br>Definition: (1) TBI (open or closed) of at least moderate severity<br><br>Method of identification: Documented by 1 or more of the following in the medical record: Initial Glasgow Coma Scale score less than 13, loss of consciousness at least 1	N=4 (40 %). 1 sustained TBI through gunshot wound, 3 from blunt assault.<br><br>Gunshot wound, Blunt assault	Intervention: ASMT<br><br>Team: 3 research therapists ((2 doctoral-level psychologists and 1 master's-level clinician)<br><br>Outcome: Participants reported a significantly lower level of anger 1 to 3 weeks after completing the ASMT compared to pretreatment.	Barrier: The most frequently cited problem was with memory for assigned tasks. For example, several participants reported difficulty in remembering or initiating completion of the logs documenting critical incidents related to anger.<br><br>Facilitator: NR<br><br>Gap:

Supplementary File 2

Charting Table

Study (Author, Year, Country)	Study Design & Objective	Study Sample	TBI Status	Violence Status	Rehabilitation Intervention, Team, Outcome	TBI-Specific Barriers, Facilitators, and Gaps
	management training (ASMT).		hour, disorientation at least 24 hours (intoxication/sedation ruled out as cause of any of these findings), and/or positive neuroimaging findings consistent with TBI; (2) at least 6 months since injury<br><br>Severity: At least moderate severity<br><br>Time since injury: At least 6 months since injury			• Program could be better tailored to such cultural factors bearing on the expression of anger<br>• Need for larger sample size and longer follow-up interval
**Horan et al., 2019**<br><br>**South Africa**	Case study<br><br>Case description of a unique case of incomplete (Locked in Syndrome) LIS after supratentorial injury. Initial ICU care and early rehabilitation likely played a major role in the full recovery of this patient.	N = 1<br><br>Age: 26<br><br>Sex/Gender [not specified]*: Woman<br><br>Race/ethnicity: NR<br><br>Other: N/A	N = 1 (100 %)<br><br>Definition: NR<br><br>Method of identification: Glasgow Coma Scale, CT scan<br><br>Severity: NR<br><br>Time since injury: NR	N = 1 (100 %)<br><br>Gunshot to the left suboccipital area, with supratentorial extension	Intervention: Early daily physiotherapy and occupational therapy rehabilitation, speech therapy assistance<br><br>Team:<br>• Physiotherapist<br>• Occupational therapist<br>• Speech therapist<br><br>Outcome:<br>• The patient first sat out of bed in the sixth week.<br>• By week 7 postinjury, she could weight-bear with assistance, and started taking assisted steps the following week.<br>• Within the sixth week, she started to verbalize, and by the end of week 6 she was communicating with speech therapist assistance. This	Barrier: NR<br><br>Facilitator: In this case, early diagnosis of Locked in Syndrome(LIS) was made, which is uncommon, and this allowed early intervention and physiotherapy, which we think also contributed to the rapid recovery of the patient.<br><br>Gap: NR

Supplementary File 2

Charting Table

Study (Author, Year, Country)	Study Design & Objective	Study Sample	TBI Status	Violence Status	Rehabilitation Intervention, Team, Outcome	TBI-Specific Barriers, Facilitators, and Gaps
					improved rapidly to near normal speech by the seventh week.<br>• She was discharged to a rehabilitation facility at the end of post admission week<br>• On the last follow-up visit on post injury month 4, she was ambulating with a walking frame with incomplete recovery of fine motor hand function.	
**Jackson et al., 2020**<br><br>**South Africa**	Quantitative<br><br>To evaluate the outcomes of the Neurodevelopmental Treatment (NDT) and motor relearning occupation-based approaches on physical performance and self-care among adults with TBI	N = 60<br><br>Age (mean): 36.5<br><br>Sex n%:<br>Male = 40(66.7 %)<br>Female = 20(33.3 %)<br><br>Other:	N = 60(100 %)<br><br>Definition: NR<br><br>Method of identification: NR<br><br>Severity: Mild to moderate TBI (GCS Score 9 -15) with evidence of decreased level of consciousness on admission and a present Glasgow Coma Scale (GCS) of 15/15<br><br>Time since injury: NR	30 % of participants<br><br>NR	Intervention:<br>• NDT approach - inhibitory control of abnormal movements at the same time facilitating automatic postural reactions using the therapist's hands and different techniques in goal-directed activities.<br>• Motor relearning occupation-based approach - The approach is task-oriented because it encourages the use of meaningful activities that are contextually based and incorporates active participation to achieve functional recovery and motor relearning by repetitive and intensive practice.<br><br>Team: Occupational therapist<br><br>Outcome:<br>• For occupational performance outcomes, no significant differences were found in the current study.	Barrier: NR<br><br>Facilitator: NR<br><br>Gap:<br>• All participants received other forms of therapy during this period and it is not clear what effect this may have had on their improvement.<br>• It is possible that some aspects of the individual therapists and the environment such as a treatment setting, ways of instruction and feedback might have led to some biases that were not controlled for in this study which might have affected the effect sizes.

Supplementary File 2

Charting Table

Study (Author, Year, Country)	Study Design & Objective	Study Sample	TBI Status	Violence Status	Rehabilitation Intervention, Team, Outcome	TBI-Specific Barriers, Facilitators, and Gaps
					• Significant improvement in physical performance and self-care in both Group 1 motor relearning occupation-based approach participants and Group 2 NDT approach participants.<br>• However, the motor relearning occupation-based approach was found to be significantly superior in self-care outcomes as well as some upper extremity outcomes.<br>• NDT treatment approach was found to be significantly superior in addressing joint pain domains which had a significant increase compared to the other group.	
**Kapur, N.; Gordon, D. S., 1975**<br><br>**Ireland**	Case study<br><br>To discuss a case of dysgraphia, the investigative methods which were employed, and the training scheme by which an improvement in writing performance was produced	N = 1<br><br>Age: 24<br><br>Sex/Gender: Man<br><br>Race/ethnicity: NR<br><br>Other: Labourer	N=1 (100 %)<br><br>Definition of TBI: NR<br><br>Method of identification: Radiography of the skull showed an extensive comminuted fracture and revealed numerous shot gun pellets in the scalp and brain. Extensive debridement was required.<br><br>Severity: NR<br><br>Time since injury: NR	N=1 (100 %)<br><br>Gunshot wound to the head	Intervention: Dysgraphia training - The basic method of retraining was to use the patient's repertoire of efficient responses to aid the recovery of inefficient response patterns. Within this broad approach, two general techniques were employed- providing the patient with assistance in the performance of writing, with gradual reduction of this support in subsequent training sessions; and, encouraging the patient to adopt alternative coding strategies in his production of spatial-motor responses.<br><br>Team: NR	Barrier: Patient had limited insight to his injury<br><br>Facilitator: NR<br><br>Gap: NR

Supplementary File 2

Charting Table

Study (Author, Year, Country)	Study Design & Objective	Study Sample	TBI Status	Violence Status	Rehabilitation Intervention, Team, Outcome	TBI-Specific Barriers, Facilitators, and Gaps
					Outcome: The emphasis in retraining was on the writing of lower-case letters since they are of more importance for most writing tasks. Little recovery took place in the six-month period between the discharge of the patient and his readmission for cranioplasty suggests that, left to itself, spontaneous recovery did not lead to much improvement in handwriting ability. Precision of concepts and adequacy of experimental control are often difficult to achieve in the field of retraining of psychological functions after brain damage. Consequently, methods of investigation and retraining may not be as generally applicable as in other areas of psychological inquiry.	
**Kelly et al., 2022**<br><br>**Australia**	Case series<br><br>To investigate (i) the feasibility of behaviour support interventions to reduce Inappropriate Sexual Behaviours (ISX) among the sample; (ii) the maintenance of	N = 24<br><br>Age (mean ± SD): 35.38 ± 13.74<br><br>Sex (n%)<br>Male – 19(79.2 %)<br>Female – 5(20.8 %)<br><br>Race/ethnicity: NR<br><br>Other (n%):	N=24 (100 %) experienced TBI<br><br>Definition of TBI: Adult onset (18-65), TBI, stroke or hypoxic brain injury<br><br>Method of identification: NR<br><br>Severity: NR	N=2 (8.3 %) experienced TBI through assault<br><br>Assault	Intervention: Behaviour support intervention – The behavioural intervention designs have generally been informed by applied behaviour analysis (ABA), with scheduled feedback or differential reinforcement of low rates of behaviour being the two most common techniques employed.	Barrier: Due to state funding arrangements, clients relied on “public” services, and were not eligible for private services such as that funded through insurance. Funding was scarce, and clients who did not live in the family home typically relied on public housing, disability support payments, and the public service system.

Charting Table

Study (Author, Year, Country)	Study Design & Objective	Study Sample	TBI Status	Violence Status	Rehabilitation Intervention, Team, Outcome	TBI-Specific Barriers, Facilitators, and Gaps
	behaviour changes at a follow-up time point; and (iii) the course of other challenging behaviours during the same time period.	• Preinjury criminal behaviour (nonsexual) - 4(16.7 %)<br>• Preinjury psychiatric/emotional disturbance - 9(37.5 %)<br>• Preinjury substance abuse - 6(25.0 %)<br>• Preinjury marital status<br>- Married/defacto - 14(58.3 %)<br>- Single - 7(29.2 %)<br>- Separated/divorced - 3(12.5 %)<br>• Preinjury employment status<br>- Full time - 19(79.2 %)<br>- Part time - 1(4.2 %)<br>- Unemployed - 3(12.5 %)<br>• Preinjury Living situation<br>- Alone - 2(8.3 %)<br>- Parents - 4(16.7 %)<br>- Spouse 12(50.0 %)<br>- Other family - 3(12.5 %)<br>- Friends 1 (4.2 %)<br>- Attendant care/nursing 1(4.2 %)<br>*Missing data – 1	Time since injury: NR		Team: State registered psychologists<br><br>Outcome: Significant decline in inappropriate sexual behaviors from baseline to closure that was maintained at follow-up	Facilitator: NR<br><br>Gap:<br>• Study was not controlled and the inappropriate sexual behaviours may have declined for reasons independent of the intervention.<br>• Therapists delivering the intervention collected the Overt Behaviour Scale assessment data at the three time points, giving rise to a potential risk of bias in their approach to measuring possible behaviour change.<br>• Behaviours were measured using probes at key timepoints, rather than continuous monitoring which would better reflect behaviour changes across time.

Supplementary File 2

Charting Table

Study (Author, Year, Country)	Study Design & Objective	Study Sample	TBI Status	Violence Status	Rehabilitation Intervention, Team, Outcome	TBI-Specific Barriers, Facilitators, and Gaps
**Killington et al., 2015**<br><br>**Australia**	Qualitative<br><br>To investigate how a vestibular deficit following an ABI affects an individuals' quality-of-life	N = 9<br><br>Age range: 19-61 years old<br><br>Sex/Gender [not specifies]*:<br>Did not identify total number of males/men or females/women but referred to participants as he/him or she/her.<br><br>She/her = 5<br>He/him – 4<br><br>Race/ethnicity: NR<br><br>Other: Intersection with mental health - Although P5 felt that his family and close friends understood and accepted his symptoms, he felt others suspected him of 'lying about things' and he was glad that he could show scars he had to prove that he had indeed sustained an ABI. In addition, he was able to explain his condition to others after receiving vestibular rehabilitation, which he felt validated his difficulties and ongoing symptoms. P5 felt 'panicky' when he became dizzy if he was alone. This	N=9 (100 %)<br><br>Definition: NR<br><br>Method of identification: Had an acquired brain injury within the last 24months,had a vestibular deficit as assessed by their treating physiotherapist or medical officer while receiving ambulatory rehabilitation through the South Australia Brain Injury Rehabilitation Services, had cognition and communication skills to enable participation<br><br>Severity: NR<br><br>Time since injury: Last 24 months	N=1 (11.1 %)<br><br>Assault	Intervention: Specialist neurologically trained physiotherapist, working in Brain Injury Rehabilitation - The therapist is required to guide each patient to use adaptive processes that suit their individual experience, training, lifespan and cognitive style and other individual characteristics before and after their vestibular deficit. Each patient should be supported to use different sensory, motor and cognitive strategies to adapt to their vestibular deficit.<br><br>Team: Physiotherapist or medical officer<br><br>Outcome: After being referred to the specialist brain injury community rehabilitation program, the physiotherapist could explain what was going on and help him and he reported feeling 'very good about myself, happy, coping with everyone at home'. P5's main symptom was dizziness due to the vestibular impairment in conjunction with increased levels of frustration and difficulty filtering stimuli, which resulted in reduced tolerance to loud music and difficulty managing family life.	Barrier: "He reported that he saw many doctors after his assault and ABI 12 months previously, but felt that his symptoms were not being addressed. He said that he thought the doctors knew why he was dizzy, but 'they just seemed to think it would go away after a while'. He said he initially received no practical help 'just checking, checking, checking." (ref to P5)<br><br>Facilitator:<br>• Validation of ongoing symptoms<br>• Timely diagnosis, explanation and education, and implications of condition<br><br>Gap: Diagnosis of vestibular deficits can be complex resulting in a delay of treatment post ABI which can also lead to misdiagnosis.

Charting Table

Study (Author, Year, Country)	Study Design & Objective	Study Sample	TBI Status	Violence Status	Rehabilitation Intervention, Team, Outcome	TBI-Specific Barriers, Facilitators, and Gaps
		young man, who had previously enjoyed his independence, preferred now to have people around him if he ventured away from home to provide reassurance and to ensure that he felt safe.				

Supplementary File 2

Charting Table

Study (Author, Year, Country)	Study Design & Objective	Study Sample	TBI Status	Violence Status	Rehabilitation Intervention, Team, Outcome	TBI-Specific Barriers, Facilitators, and Gaps
**Kim et al., 2013**<br><br>**Canada**	Retrospective cohort study<br><br>To determine (a) whether differences exist in rehabilitation outcomes between intentional and unintentional TBI populations and (b) whether TBI from assault is a predictor of community integration following in patient rehabilitation	N=243<br><br>Age range: 30 – 34<br><br>Sex:<br>Male -190<br>Female - 53<br><br>Race/ethnicity: NR<br><br>Other (n%):<br>• Language (English) - 235(96.7 %)<br>• Vocational status (Employed) - 150(61.7 %)<br>• Alcohol/drug abuse history - 73(30 %)<br>• Living arrangement<br>- Living with others - (82.3 %)<br>- Urban/rural dwelling (urban) - 177(72.8 %)<br>- Discharged home - 207(85.2 %)	N=243 (100 %) sustained TBI<br><br>Definition: TBI from physical assault, excluding self-inflicted injuries<br><br>Method of identification: NR<br><br>Severity: NR<br><br>Time since injury: NR	24(9.9 %) had sustained TBI from physical assault<br><br>Physical assault, interpersonal violence, war	Intervention: Inpatient rehabilitation<br><br>Team: Occupational therapists<br><br>Outcome: NR	Barrier: NR<br><br>Facilitator: NR<br><br>Gap: Researchers do not have all desirable measures in the existing databases to fully investigate TBI from intentional causes, such as level of education, criminality, ethnicity, and previous level of community integration

Supplementary File 2

Charting Table

Study (Author, Year, Country)	Study Design & Objective	Study Sample	TBI Status	Violence Status	Rehabilitation Intervention, Team, Outcome	TBI-Specific Barriers, Facilitators, and Gaps
**Lind et al., 2016**<br><br>**France**	Retrospective study<br><br>To report long-term neurological, cognitive, behavioral and academic outcomes, ongoing treatments and/or rehabilitation, several years after Abusive Head Trauma (AHT) diagnosis, and factors associated with outcome.	N = 47<br><br><u>Age (mean)</u>: at time of injury = 5.7 months<br><br><u>Sex-ratio</u> (boys/girls) 3.7<br><br><u>Race/ethnicity</u>: NR<br><br><u>Other:</u><br><br>Mothers' education level equal or superior to graduation from high school [n (%)] 34(72 %)	N=47 (100 %: TBI through abusive head trauma)<br><br><u>Definition:</u> NR<br><br><u>Method of identification:</u> NR<br><br><u>Severity</u>: Extremely severe neurological impairment (GOS IV)<br><br><u>Time since injury:</u> NR	N=47 (100 %)<br><br>Abusive head trauma	<u>Intervention:</u> Rehabilitation unit<br><br><u>Team:</u> speech and language therapy, physiotherapy, occupational therapy, psychometricians, neurocognitive psychologists<br><br><u>Outcome:</u><br>• Less than 10 % of the sample had returned to normal life according to the GOS.<br>• After a median delay of 8 years post-injury, a large proportion suffered medical, visual, neurological, cognitive (including language difficulties) and behavioral impairments	<u>Barrier:</u><br>• Lower educated mothers may be unable to seek adequate support and resources for their child<br>• Poor family stability, childcare and low parental socioeconomic status can affect outcomes<br><br><u>Facilitator:</u> NR<br><br><u>Gap:</u> Future research is required to tease out the effect of wider indicators of socio-economic status, such as parental education (used here as a proxy for SES), but also family functioning, coping skills, etc.

Supplementary File 2

Charting Table

Study (Author, Year, Country)	Study Design & Objective	Study Sample	TBI Status	Violence Status	Rehabilitation Intervention, Team, Outcome	TBI-Specific Barriers, Facilitators, and Gaps
**MacDonald et al., 2001**<br><br>**United States**	Case study<br><br>Presents the clinical findings and treatment of a six-month-old infant with Shaken Baby Syndrome who received inpatient occupational therapy	N = 1<br><br><u>Age</u>: 6-month-old<br><br><u>Sex/Gender</u> [not specified]*: Male<br><br><u>Race/ethnicity</u>: NR<br><br><u>Other:</u> Unmarried teenage parents (age 16 and 17), economic strain (working opposite shifts), difficulty soothing crying infant	n = 1 (100 %)<br><br><u>Definition:</u> "In order to be labeled Shaken Baby Syndrome the shaking must be of such a force that even a lay person would recognize the act as dangerous [...] When an infant is shaken, the brain jerks back and forth in the skull, resulting in a coup and counter coup injury to the brain. Blood vessels in and around the brain are damaged and can begin to bleed into the brain causing further damage."<br><br><u>Method of identification</u>: CT scan revealed a previous blunt insult to the head, subarachnoid hemorrhaging, and various lesions in the brain.<br><br><u>Severity</u>: NR<br><br><u>Time since injury</u>: NR	n = 1 (100 %)<br><br>Shaken Baby Syndrome	<u>Intervention:</u> Occupational therapy sessions to promote age-appropriate development<br><br><u>Team:</u><br>• Occupational therapist<br>• Physical therapist<br>• Speech therapist<br><br><u>Outcome:</u><br>• Child placed in legal guardianship of mother's aunt. family training and education provided<br>• Family was educated on the psychosocial and physical deficits that resulted from the brain dam- age.<br>• The occupational therapist validated the common feelings of frustration that arise when caring for an infant, especially an inconsolable infant.	<u>Barrier</u>: In the case of many infants with Shaken Baby Syndrome, the legal guardian and/or discharge destination is unknown. Many times, when an infant is placed in a foster home, the infant will be discharged to the foster family without any training or education occurring.<br><br><u>Facilitator:</u> NR<br><br><u>Gap</u>: NR

Supplementary File 2

Charting Table

Study (Author, Year, Country)	Study Design & Objective	Study Sample	TBI Status	Violence Status	Rehabilitation Intervention, Team, Outcome	TBI-Specific Barriers, Facilitators, and Gaps
**McIntosh, C.; James, A., 2018**<br><br>**United Kingdom**	Case study<br><br>This article considers the complexities of neuropsychological assessment and rehabilitation in brain injury when the client is illiterate, is from a foreign culture with English as a second language, and reports highly atypical childhood feral experiences prior to injury.	N = 1<br><br>Age: 63, however injuries were received during teen years.<br><br>Sex/Gender [not specified]*: Female<br><br>Race/ethnicity [not specified]**: Hispanic<br><br>Other: Held role within the family and employment as a chef	n = 1 (100 %), patient MC.<br><br>Definition: NR<br><br>Method of identification: CT and MRI scan<br><br>Severity: NR<br><br>Time since injury: Teenage years	n = 1 (100 %), patient MC.<br><br>MC reported a history of minor head injury sustained as a teenager. She was regularly beaten and sustained injuries to the head, some of which she reported led to periods of altered consciousness.	Intervention: Specialist residential neuropsychological rehabilitation – includes family therapy<br><br>Team: Support worker<br><br>Outcome:<br>• Greatly reduced pragmatic difficulties<br>• Increased ability to engage in reciprocal interactions.<br>• Reduced functional cooking errors<br>• Able to cook more independently at home, requiring no supervision	Barrier: Many people with cognitive impairment in hospital will be assessed with brief screening measures by healthcare professionals without expertise in neuropsychology. In cases with other cultural backgrounds this often can lead to misdiagnosis or inaccurate treatment.<br><br>Facilitator: Behavioural observations, functional assessments and information from her family were all considered with test interpretation and for formulation.<br><br>Gap: The tests used likely under-represented the subjects' true abilities due to potential cultural barriers.

Supplementary File 2

Charting Table

Study (Author, Year, Country)	Study Design & Objective	Study Sample	TBI Status	Violence Status	Rehabilitation Intervention, Team, Outcome	TBI-Specific Barriers, Facilitators, and Gaps
**Neumann et al., 2015**<br><br>**United States**	RCT<br><br>To examine the effectiveness of 2 affect recognition interventions (Faces and Stories) in people with a traumatic brain injury.	N = 203 participants with moderate to severe traumatic brain injury were screened; 71 were eligible and randomized to the Faces (n = 24), Stories (n = 23), and Control interventions (n = 24)<br><br>Age:<br>Faces intervention n = 24 (mean age 41)<br>Stories intervention n = 23 (mean age 41.5) Control intervention n = 24 (mean age 39.5)<br><br>Sex:<br>Faces intervention n = 24 (96 % male)<br>Stories intervention n = 23 (78 % male)<br>Control intervention n = 24 (67 % male)<br><br>Ethnicity:<br>Faces intervention n = 24 (87 % White, 13& African American).<br>Stories intervention n = 23 (78 % White, 13 % African American, 4 % Hispanic, 4 % Other).<br>Control intervention n = 24 (71 % White, 21 % African American, 4 % Maori, 4 % Other).	N= 95 % severe TBI<br><br>Definition: NR<br><br>Method of identification: GCS score at time of injury, post traumatic amnesia, loss of consciousness obtained via medical records or self/caregiver report when medical records were not available<br><br>Severity: Moderate to severe<br><br>Time since injury: NR	Faces intervention n = 24 (9 % experienced assault)<br>Stories intervention n = 23 (9 % experienced assault)<br>Control intervention n = 24 (8 % experienced assault)<br><br>Assault	Intervention: Computer-based training - All 3 interventions were a one-on-one computer assisted treatment facilitated by a therapist, who received approximately 16 hours of training in administering the intervention. Therapists had either completed or were currently enrolled in a graduate-level healthcare-related program and/or had experience working with patients with TBI<br><br>Team: Therapists (not specified)<br><br>Outcome:<br>• Participants who received the Faces Intervention were significantly better at recognizing facial expressions posttreatment than participants who received the Control intervention.<br>• Participants who received the Faces Intervention did no better than those who received the control intervention on inferring emotions from stories and empathy measures.	Barrier: Inability to access medical records and reliance on patient/caregiver for information on severity<br><br>Facilitator: NR<br><br>Gap: NR

Supplementary File 2

Charting Table

Study (Author, Year, Country)	Study Design & Objective	Study Sample	TBI Status	Violence Status	Rehabilitation Intervention, Team, Outcome	TBI-Specific Barriers, Facilitators, and Gaps
		Other: Average years of education for each sample:<br>Faces intervention (12.3)<br>Stories intervention (13.2)<br>Control intervention (12.6)				

Supplementary File 2

Charting Table

Study (Author, Year, Country)	Study Design & Objective	Study Sample	TBI Status	Violence Status	Rehabilitation Intervention, Team, Outcome	TBI-Specific Barriers, Facilitators, and Gaps
**Neumann et al., 2017**<br><br>**United States**	Quantitative<br><br>To examine the acceptability and initial efficacy of an emotional self-awareness treatment at reducing alexithymia and emotion dysregulation in participants with traumatic brain injury (TBI)	N = 17<br><br>Age: Mean age= 46.12<br><br>Sex n%:<br>male – 13(76.5 %)<br>female- 4(23.5 %)<br><br>Race (n%):<br>White 16 (94.1 %)<br>Other 1 (5.9 %)<br><br>Other:<br>Education mean – 14.06 years	N = 17(100 %)<br><br>Definition: NR<br><br>Method of identification: "as defined by at least one of the criteria outlined by the Mayo classification system for TBI: A Glasgow Coma Scale score of less than 13 (at the time of injury), posttraumatic amnesia 24 hours or more, loss of consciousness 30 minutes or more, or abnormal neuroimaging results"<br><br>Severity: Moderate to severe<br><br>Time since injury: NR	N=1 (8 % experienced assault)<br><br>Assault	Intervention: Psychoeducation and skill-building exercises<br><br>Team: NR<br><br>Outcome:<br>• Significant improvements in participants' emotional self-awareness and ability to describe and differentiate emotions immediately after treatment and 2 months later<br>• 62 % of participants reduced the categorical severity of their alexithymia<br>• Initial improvements for anxiety, positive affect and emotion dysregulation<br>• Trend toward reduction in anger	Barrier:<br>• Transportation challenges<br>• Communication problems that prohibited participation<br><br>Facilitator: NR<br><br>Gap: The small sample size, although adequate to determine effect size, reduces generalization of these results to the larger TBI population.

Supplementary File 2

Charting Table

Study (Author, Year, Country)	Study Design & Objective	Study Sample	TBI Status	Violence Status	Rehabilitation Intervention, Team, Outcome	TBI-Specific Barriers, Facilitators, and Gaps
**Nikopoulos et al., 2013**<br><br>**United Kingdom**	Case study<br><br>To examine the efficacy of video modelling in emerging speech in an adult male with TBI caused by an assault	N = 1<br><br>Age: 34<br><br>Sex/Gender [not specified]*: Male<br><br>Race/ethnicity: NR<br><br>Other: NR	N = 1 (100 %)<br><br>Definition: TBI present with impairments ranging from mild-to-severe that can affect a variety of domains including cognition, sensory-motor, adaptive behaviours, social skills and communication<br><br>Method of identification: NR<br><br>Severity: NR<br><br>Time since injury: NR	N = 1 (100 %)<br><br>Assault	Intervention: Video modelling<br><br>Team: Speech language pathologist<br><br>Outcome: Video modelling can promote the performance of previously learned behaviours related to speech, but more significantly it can facilitate the generalization of this verbal behaviour across untrained words.	Barrier: NR<br><br>Facilitator: NR<br><br>Gap: Struggle to find balance between individual and group rehab treatment as groups can mask the individual characteristic of someone with TBI

Supplementary File 2

Charting Table

Study (Author, Year, Country)	Study Design & Objective	Study Sample	TBI Status	Violence Status	Rehabilitation Intervention, Team, Outcome	TBI-Specific Barriers, Facilitators, and Gaps
**Obiano et al., 2022**<br><br>**United Kingdom**	Prospective cohort<br><br>To investigate the incidence of persistent post-concussion symptoms and possible predictors of long-term disability focusing on demographic, injury, and psychological factors	N = 1131<br><br>Age mean (SD): 45.5 (17–83)<br><br>Gender:<br>Male - 779 (69 %)<br>Female - 352 (31 %)<br><br>Ethnicity:<br>White - 1043 (92 %)<br>Non-white - 88 (8 %)<br><br>Other n(%):<br>• No home support - 449 (40 %)<br>• With home support - 682 (60 %)<br><br>Co-morbidity<br>• None 842- (74 %)<br>• Yes 289 - (26 %)<br><br>Working at the time of injury<br>• Yes 966 - (85 %)<br>• No 165 - (15 %)<br><br>Alcohol intoxication<br>• No 839 - (74 %)<br>• Yes 292 - (26 %)	N = 1131 (100 % TBI)<br><br>Definition: NR<br><br>Method of identification: NR<br><br>Severity: NR but mentioned that severity was classified through the GCS in the ED and classified as mild, moderate, and severe<br><br>Time since injury: NR	n = 214 (19 %)<br><br>Assault	Intervention: Neurorehabilitation Brain Injury Clinic<br><br>Team:<br>• Brain injury specialist doctor<br>• Clinical nurse specialist<br>• Brain injury social worker<br><br>Outcome: NR	Barrier: NR<br><br>Facilitator: NR<br><br>Gap: NR

Supplementary File 2

Charting Table

Study (Author, Year, Country)	Study Design & Objective	Study Sample	TBI Status	Violence Status	Rehabilitation Intervention, Team, Outcome	TBI-Specific Barriers, Facilitators, and Gaps
**Olsen et al., 2022**<br><br>**Norway**	Mixed methods<br><br>To identify candidate structural brain measures with relevance for rehabilitation of cognitive control (executive) function after TBI	N = 28<br><br>Age (mean): 40.5<br><br>Sex (n%):<br>Male – 19(67.9 %)<br>Female – 9(32.1 %)<br><br>Race/ethnicity: NR<br><br>Other:<br>Mean education (years): 13.43	N = 28(100 %)<br><br>Definition: NR<br><br>Method of identification: Glasgow Coma Scale (GCS) and radiological findings (MRI/CT)<br><br>Severity: All included patients had complicated mild, moderate or severe TBI<br><br>Time since injury: NR	N=2 (7.1 %)<br><br>Injury mechanism referred to as 'violence'	Intervention: The participants were randomized to either Goal Management Training (GMT) or the Brain Health Workshop (BHW)<br><br>Team: NR<br><br>Outcome:<br>• Regional brain volume at baseline was significantly associated with treatment outcome.<br>• Measures of brain structure obtained before treatment are associated with cognitive rehabilitation outcomes. Both positive and negative associations between outcome and regional brain volume in a wide range of anatomical locations were observed. The most pronounced associations between larger TBM-based regional brain volume and positive outcome were found in midline fronto-parietal cortical regions, including the anterior and posterior cingulate cortices which are known to be key areas for cognitive control processing in the general population and functionally altered after TBI.	Barrier: NR<br><br>Facilitator: NR<br><br>Gap: The important clinically relevant question of what works for whom, and why, in the context of cognitive rehabilitation after TBI is still largely unanswered.

Supplementary File 2

Charting Table

Study (Author, Year, Country)	Study Design & Objective	Study Sample	TBI Status	Violence Status	Rehabilitation Intervention, Team, Outcome	TBI-Specific Barriers, Facilitators, and Gaps
**Poggel et al., 2001**<br><br>**Germany**	Case study<br><br>Over a period of more than 3 years, changes in visual and neuropsychological functions were examined in a patient with a visual field defect caused by a cerebral gunshot lesion.	N = 1<br><br>Age: 29<br><br>Sex/Gender [not specified]*: Male<br><br>Race/ethnicity: NR<br><br>Other: NR	N = 1 (100 %)<br><br>Definition: Attacked and shot in the back of his head<br><br>Method of identification: NR<br><br>Severity: NR<br><br>Time since injury: NR	N = 1 (100 %)<br><br>Assault and gunshot	Intervention: Visual restitution training<br><br>Team: Neuropsychology<br><br>Outcome:<br>• Patient's spontaneous recovery ended only 16 months after lesion.<br>• Spontaneous improvement was striking in that he regained almost 2/3 of his visual field although average recovery in patients with complete cortical blindness usually amounts to about 28 %.	Barrier: NR<br><br>Facilitator: NR<br><br>Gap: NR

Supplementary File 2

Charting Table

Study (Author, Year, Country)	Study Design & Objective	Study Sample	TBI Status	Violence Status	Rehabilitation Intervention, Team, Outcome	TBI-Specific Barriers, Facilitators, and Gaps
**Raszka et al., 2018**<br><br>**Poland**	Case report<br><br>This work presents the description of the case, 35 who suffered from cranial-cerebral injury as a result of the gunshot wound in vague circumstances and chosen nursing problems of the patient after the gunshot wound	N = 1<br><br>Age: 35<br><br>Sex: Male<br><br>Race/ethnicity: NR<br><br>Other: Admitted in a state of alcohol intoxication	N = 1 (100 %)<br><br>Definition: Cranial-cerebral injury as a result of the gunshot wound CT<br><br>Method of identification: NR<br><br>Severity: NR<br><br>Time since injury: NR	N = 1 (100 %)<br><br>Gunshot wound to the head	Intervention: Long-term rehabilitation<br><br>Team:<br>• Occupational therapist<br>• Physical therapist<br>• Psychologist<br>• Psychiatrist<br>• Nurse<br><br>Outcome:<br><br>Status at discharge:<br>• The patient is conscious, with right-sided paresis, oriented to his own person, place and time, calm.<br>• Verbal contact maintained (aphasia withdraws).<br>• The patient with a deficit in self-care and self-nursing; rehabilitated, upright.<br>• He requires assistance in selected activities of everyday life, strives to be independent in the performance of simple activities.<br>• He controls the physiological needs.<br>• Discharged home in good general condition.<br>• Scheduled for admission to the Rehabilitation Ward to continue the improvement.	Barrier: NR<br><br>Facilitator: An important role is played by the assessment of the deficit in the scope of self-care as well as possible problems that may arise from the general condition of the patient and the assessment of the family's efficiency and knowledge in the scope of care activities.<br><br>Gap: NR

Supplementary File 2

Charting Table

Study (Author, Year, Country)	Study Design & Objective	Study Sample	TBI Status	Violence Status	Rehabilitation Intervention, Team, Outcome	TBI-Specific Barriers, Facilitators, and Gaps
**Risen et al., 2014**<br><br>**United States**	Retrospective review<br><br>To compare clinical features and functional outcomes of age- and sex-matched children with abusive and nonabusive head trauma receiving inpatient rehabilitation.	N = 48<br><br>Age:<br>Mean: 20<br>Range: 2-51 months<br><br>Sex (n%)<br>Female – 17(61 %)<br><br>Race/ethnicity: NR<br><br>Other: NR	N = 48 (100 % TBI patients)<br><br>Definition: Abusive head trauma<br><br>Method of identification: Acute care medical records<br><br>Severity: NR<br><br>Time since injury: NR	N =28 (58.3 %) sustained abusive head trauma<br><br>Abusive head trauma	Intervention: Inpatient pediatric rehabilitation<br><br>Team: NR<br><br>Outcome:<br>• Functional impairment upon admission to inpatient rehabilitation was comparable, and functional gains during inpatient rehabilitation were similar between groups.<br>• More children with nonabusive than with abusive head trauma attained independent ambulation and expressive language after discharge from rehabilitation; the difference was no longer significant when only children aged >12 months at injury were examined. There was variability in delay to obtain these skills and in the quality of gained skills in both groups.	Barrier: NR<br><br>Facilitator: NR<br><br>Gap:<br>• Unable to evaluate long-term outcomes in cognition and behavior due to inconsistent documentation of these skills in the available records.<br>• Also, unable to gather complete data on family and socioeconomic factors that have also been shown to influence outcome after TBI in children

Supplementary File 2

Charting Table

Study (Author, Year, Country)	Study Design & Objective	Study Sample	TBI Status	Violence Status	Rehabilitation Intervention, Team, Outcome	TBI-Specific Barriers, Facilitators, and Gaps
**Rojas-Ramirez et al., 2016**<br><br>**United States**	Case report<br><br>A report presenting a case of Short-lasting unilateral neuralgiform headache with conjunctival injection and tearing (SUNCT) secondary to head and neck trauma	N = 1<br><br>Age: 57<br><br>Sex/Gender:[not specified]* Female<br><br>Race/ethnicity: NR<br><br>Other: NR	N = 1(100 %)<br><br>Definition: NR<br><br>Method of identification: Neuropsychometric evaluation<br><br>Severity: Mild<br><br>Time since injury: NR	N = 1(100 %)<br><br>Violent assault	Intervention: Physiotherapy, psychotherapy<br><br>Team:<br>• Clinical psychologist<br>• Behavioural medicine specialists<br>• Psychologist<br>• Rehabilitation psychologist<br>• Neurologist<br>• Physiotherapist<br><br>Outcome: NR	Barrier: NR<br><br>Facilitator: NR<br><br>Gap: NR

Supplementary File 2

Charting Table

Study (Author, Year, Country)	Study Design & Objective	Study Sample	TBI Status	Violence Status	Rehabilitation Intervention, Team, Outcome	TBI-Specific Barriers, Facilitators, and Gaps
**Rotarescu, V.; Ciurea, A. V., 2008**<br><br>**Romania**	Longitudinal and transversal study<br><br>To evaluate the effects of Mild Head Injury in children not only in terms of impairment, but also in terms of disability, handicap and quality of life	N = 96<br><br>Age (mean): 10.46<br><br>Sex:<br>Male = 68(70.8 %)<br><br>Ethnicity: Romanian<br><br>Other: NR	N = 96 (100 % children with mild head injury)<br><br>Definition: NR<br><br>Method of identification: NR<br><br>Severity: most TBIs are mild with loss of consciousness of five minutes or less, posttraumatic amnesia for less the 12 hours, and an initial score on Glasgow or Children Coma Scale of 13 to 15<br><br>Time since injury: NR	N =2 (2.1 %)<br><br>Assault	Intervention: Psychotherapy<br><br>Team:<br>NR<br><br>Outcome: NR	Barrier:NR<br><br>Facilitator: NR<br><br>Gap: NR
**Sample et al., 2012**<br><br>**United States**	Qualitative<br><br>To learn about and come to an understanding of the recovery process and outcomes experienced by the survivors of the 1995 Oklahoma City bombing, who sustained a traumatic brain injury (TBI) along with other injuries in the blast	N = 46<br><br>Age:<br>Life Stages: Adult (17), Parent (6), Child (3)<br><br>Gender:<br>Male 11 Female 9<br><br>Race:<br>• Caucasian/Non-Hispanic 14 (70 %)<br>• African American 4 (20 %)<br>• Other 2 (10 %)<br><br>Other:<br>Life Stage<br>Adult 17	N = 20 (44 %)<br><br>Definition: TBI secondary to blast<br><br>Method of identification: NR<br><br>Severity: NR<br><br>Time since injury: NR	N =20 bombing survivors(44 %)<br><br>Oklahoma City bombing	Intervention: occupational therapy, physical therapy and some speech therapy, therapeutic horse back riding, support groups, therapy<br><br>Team: Occupational therapist, physical therapist, speech therapist<br><br>Outcome: NR	Barrier:<br>Cost of mental health treatment<br><br>Facilitator: Support systems<br><br>Gap: NR

Supplementary File 2

Charting Table

Study (Author, Year, Country)	Study Design & Objective	Study Sample	TBI Status	Violence Status	Rehabilitation Intervention, Team, Outcome	TBI-Specific Barriers, Facilitators, and Gaps
		Parent 6<br>Child 3<br><br>Occupational status when interviewed<br>Employed 12<br>Volunteering (on disability) 1<br>Secondary School Students 3<br>Seeking Employment 1<br>Retired (medical or regular) 3				
**Schroy et al., 2012**<br><br>**United States**	Case study<br><br>To highlight the need to make resources available to the patient, and to stress the importance of a patient-centered approach	N = 1<br><br>Age: 40 years of age at time of study; Approximately 39 years of age when assault occurred<br><br>Sex/Gender [not specified]*: Male<br><br>Race/ethnicity: NR<br><br>Other:<br>• Employed in sales and his duties included managing sales territories and conducting conference and sales calls. He never	N = 1(100 %)<br><br>Definition: Assault by an individual with a baseball bat<br><br>Method of identification: NR<br><br>Severity: NR<br><br>Time since injury: TBI sustained in 2009	N = 1(100 %)<br><br>Assault by an individual with a baseball bat	Intervention: Computerized auditory training program called "I Hear What You Want" (2 1-hour visits per week for 6 weeks; 5 activities per session focused on meaning-based tasks-concentrated on different tasks per activity)<br><br>Team:<br>• Hearing aid services; psychosocial and vocational services<br>audiologist<br>• Referred to local hospital Deaf Services Team which provides clinical case management, psychiatric and	Barrier:<br>• Due to traumatic events, he needs were initial psychosocial than audiologic<br>• Difficult to acknowledge and undertake steps to become contributing member of society due to trauma experienced<br>• Did not want to engage in service provided as felt able to do it on his own<br>• Did seek employment on his own but took a longer period of time and was not completely satisfied with position<br><br>Facilitator:<br>• Bonded well with audiologist

Supplementary File 2

Charting Table

Study (Author, Year, Country)	Study Design & Objective	Study Sample	TBI Status	Violence Status	Rehabilitation Intervention, Team, Outcome	TBI-Specific Barriers, Facilitators, and Gaps
		returned to work because he<br>was no longer able to talk on the telephone and felt he would no longer be able to manage the communication necessary to perform his job, he consequently lost his job.<br>• The loss of employment led to the loss of his residence and possessions. At one point, he was forced to live in his car.			psychological services and if additional services needed<br><br><u>Outcome:</u><br>• Improvements in his communications abilities during the 6 weeks he was enrolled in the I Hear What You Mean computerized auditory training program and during the time he spent with the audiologist in the clinic.<br>• His auditory scores improved on some tests.<br>• He became more confident and slowly began entering the social world.<br>• On an exit questionnaire following his participation in the auditory training study, he revealed this confidence as well as perceived improvements in his communication abilities.<br>• When asked how much the training improved his ability to understand spoken language he answered, “It has eased my anxiety of listening through secondary noise.”<br>• In response to the question regarding his self-confidence in conversation with family and friends following the training he answered, “I feel more comfortable having them repeat and/or asking them to talk slow and clear.”	• Receives health insurance through employment and is considering cochlear implant<br><br>Gap: Individuals with profound hearing loss are unaware of services available to them

Supplementary File 2

Charting Table

Study (Author, Year, Country)	Study Design & Objective	Study Sample	TBI Status	Violence Status	Rehabilitation Intervention, Team, Outcome	TBI-Specific Barriers, Facilitators, and Gaps
					• His Listening Self-efficacy Questionnaire score also showed perceived improvement in his communication abilities in quiet.<br>• Finally, we administered a visual-only test when Edward first arrived and before he moved home. His scores improved dramatically and changed from 15.8% on his initial test to 68.4% on his final test.	
**Schwartz et al., 2007**<br><br>**Israel**	Mixed methods<br><br>Summarizing the rehabilitation outcomes of survivors with multiple traumas as compared to survivors with non multiple traumas injuries who were treated at a rehab facility in Israel	N = 72<br><br>Age: Mean age is 30; age range (9-76 years); individuals in non terror group mean age was 33<br><br>Sex: All individuals were male (72 in terror and 72 in non terror)<br><br>Race/ethnicity: NR<br><br>Other:<br>Demographics & n (%):<br>Education terror group:<br>Student - 9 (12.7 %)<br>Elementary - 16(22.5 %)<br>Highschool - 27 (38.0 %)	N=19 (26 %)<br><br>Definition: Multiple trauma with TBI<br><br>Method of identification: NR; GCS and coma duration to assess severity<br><br>Severity: NR<br><br>Time since injury: NR	N=19 (26 %)<br><br>Survivors of terror attacks n=9.5 (50 %) of casualties were individuals employed in blue- or white-collar jobs and others were soldiers or students	Intervention: Supervised therapy daily from both physical and occupational therapists<br><br>Team:<br>• Psychiatrist<br>• Physical therapist<br>• Occupational therapist<br>• Psychologist<br><br>Outcome:<br>• Longer periods of rehabilitation for terror victims; however, they regained most activity of daily living functions similar to nonterror group	Barrier: NR<br><br>Facilitator:<br>• Being terror victims in Israel, however, means that they were supported by many public and volunteer organizations.<br>• From the outset, they were treated by psychologists and social workers and had the close support of their families and relatives.<br>• More quality treatment for terror victims, given the sensitivity of Israeli citizens to the problem of terrorism<br><br>Gap: NR

Supplementary File 2

Charting Table

Study (Author, Year, Country)	Study Design & Objective	Study Sample	TBI Status	Violence Status	Rehabilitation Intervention, Team, Outcome	TBI-Specific Barriers, Facilitators, and Gaps
		Academic - 19 (26.8 %)<br><br>Employment terror group:<br>Student - 13 (19.1 %)<br>Soldier - 13 (19.1 %)<br>Blue collar - 24 (35.3 %)<br>White collar - 15 (22.1 %)<br>Unemployed - 2 (2.9 %)<br>Pensioner - 1 (1.5 %)			• Terror victims successfully returned to their previous occupations at a similar rate to that of the nonterror group	
**Shaklai et al., 2014**<br><br>**Israel**	Prospective cohort study<br><br>To examine functional outcomes (integration into school, socioeconomical functions, work, ability to develop long-term mature relationships and establish a family) on pediatric survivors of moderate to severe TBI after 10 years	N = 77<br><br>Age: 12-18 years (13.9 %)<br><br>Gender: 70.1 % of entire sample were male<br><br>Race/ethnicity: NR<br><br>Other: 79.2 % of the sample was Jewish.	N = 77(100 %)<br><br>Definition: NR<br><br>Method of identification: GCS, CT scan<br><br>Severity: Moderate to severe<br><br>Time since injury: NR	n=8 (10.4 %)<br><br>Assault or terror attack n=8 (10.4 %)	Intervention: Tertiary rehabilitation care - Long-term community integration in pediatric TBI<br><br>Team:<br>• Medical (not specified)<br>• Nursing<br>• Allied health professionals (not specified)<br><br>Outcome:<br>• 89.6 % of patients discharged from inpatient rehab were independent self feeders<br>• 88.3 % achieved independent mobilization	Barrier: NR<br><br>Facilitator: NR<br><br>Gap: NR

Supplementary File 2

Charting Table

Study (Author, Year, Country)	Study Design & Objective	Study Sample	TBI Status	Violence Status	Rehabilitation Intervention, Team, Outcome	TBI-Specific Barriers, Facilitators, and Gaps
					• 66 of 77 (68.9 %) were integrated into regular or special educational system<br>• 79.2 % patients that attended regular education system- found to continue school, serve in army or work<br>• 43.7 % lived outside of parents' house	
**Shklovsky et al., 2016**<br><br>**Russia**	Longitudinal case study<br><br>This clinical case illustrates the compensatory brain possibilities under long-term integrated multidisciplinary treatment and neurorehabilitation with mandatory application of medical, medico-psychological, medico-pedagogical, and medico-social methods, the importance and intensity of which vary at different stages of the disease.	N = 1<br><br>Age: 18<br><br>Sex/Gender [not specified]*: Male<br><br>Race/ethnicity [not specified]**: Born in Tula - region of Russia<br><br>Other:<br>• Two siblings (brother and sister who were twins)<br>• Graduated from 9th grade of secondary school, and entered agricultural college<br>• Worked as tractor mechanical operator at a farm in family's place of residence<br>• Worked 1 year until recruited into army<br>• No neurological or somatic conditions pre TBI	N = 1 (100 %)<br><br>Definition: Massive brain injury after a gunshot wound<br><br>Method of identification: Diagnosis mentioned but method NR<br><br>Severity: NR<br><br>Time since injury: NR	N = 1 (100 %)<br><br>Gunshot	Intervention: Neurorehabilitation<br><br>Team:<br>• Drug treatment<br>• Neuropsychological diagnostics and programming<br>• Neurolinguistic methods<br>• Psychotherapy<br>• Logotherapy<br>• Neurosensory training<br>• Ergotherapy<br><br>Outcome:<br>• Significant qualitative improvement in the motor, cognitive, and emotional domains<br>• Degree of verbal deficit changed from severe to moderate<br>• Able to get degree at a special rehabilitation centre for people with disabilities<br>• Regained ability to live independently	Barrier: NR<br><br>Facilitator:<br>• Prolonged rehab<br>• Multidisciplinary comprehensive treatment and rehab<br><br>Gap: NR

Supplementary File 2

Charting Table

Study (Author, Year, Country)	Study Design & Objective	Study Sample	TBI Status	Violence Status	Rehabilitation Intervention, Team, Outcome	TBI-Specific Barriers, Facilitators, and Gaps
**Solumsmoen, S.; Kelsen, J., 2018**<br><br>**Denmark**	Case reports<br><br>To show an example of a good treatment and rehabilitation course after penetrating head trauma	N = 1<br><br>Age: 31<br><br>Sex/Gender [not specified]*: Male<br><br>Race/ethnicity: NR<br><br>Other: NR	N = 1(100 %)<br><br>Definition: Shot in the right frontal region of the head with a low caliber projectile fired at close range<br><br>Method of identification: NR<br><br>Severity: NR<br><br>Time since injury: NR	N = 1(100 %)<br><br>Shot with low caliber projectile	Intervention: Interdisciplinary neurorehabilitation<br><br>Team: NR<br><br>Outcome:<br>• Discharged to own home after 4 months of interdisciplinary neurorehabilitation<br>• A year post injury - able to return to his previous position (different function and reduced time)<br>• A year and a half post injury - continued to have challenges in the form of dysarthria, reduced overview when performing several tasks simultaneously and in social contexts where he had to interact with several people at once. There was still slight difficulty steering his right arm, but he had learned to use his left hand through rehabilitation. Despite the extent of the primary injury, he returned to work 37 hours a week.	Barrier: NR<br><br>Facilitator: NR<br><br>Gap: NR

Supplementary File 2

Charting Table

Study (Author, Year, Country)	Study Design & Objective	Study Sample	TBI Status	Violence Status	Rehabilitation Intervention, Team, Outcome	TBI-Specific Barriers, Facilitators, and Gaps
**Stewart, I & Alderman, N, 2010**<br><br>**United Kingdom**	Case study<br><br>To consider the utility of a range of operant-derived ABI interventions specifically with regard to behaviour whose prime function is avoidance or escape from rehabilitation activities	N = 1<br><br>Age: 39 years of age; 31 years of age when sustained TBI<br><br>Sex/Gender [not specified] *: Male<br><br>Other: N/A	N = 1(100 %)<br><br>Definition: Subarachnoid haemorrhage, diffuse oedema and damage to the temporal and frontal lobes. A magnetic resonance imaging scan confirmed.<br>widespread cerebral atrophy.<br><br>Method of identification: CT scan, MRI scan<br><br>Severity: NR<br><br>Time since injury: NR	N = 1(100 %)<br><br>Assault	Intervention: Specialized neurorehabilitation service for assessment and rehabilitation; behavioral intervention<br>• Errorless learning and chaining to determine routine; complete tasks in correct order; • Differential reinforcement of incompatible behaviour strengthen frequency/duration of behaviours that are incompatible with those targeted treatment<br>• Use tokens to tangible reinforcers<br>• "time out on the spot" from positive reinforcement used or reduce likelihood of strengthening aggression and non-cooperation by not responding by staff differential reinforcement of low rates of responding (reinforcement given to successively lower rates of a target behaviour - reinforce at end of a specified time period which target behaviour<br>• Situational time out and sustained verbal prompting (use of a particular variant of time-out is not new-ensure social reinforcement was not contingent to reinforce or maintain aggression<br><br>Team: Rehabilitation professionals (not specified)	Barrier:<br>• Demands of scheduling program<br>• Need more staff to implement such a programme - balance by managers with the probability that better clinical outcomes achieved<br><br>Facilitator: NR<br><br>Gap: NR

Supplementary File 2

Charting Table

Study (Author, Year, Country)	Study Design & Objective	Study Sample	TBI Status	Violence Status	Rehabilitation Intervention, Team, Outcome	TBI-Specific Barriers, Facilitators, and Gaps
					Outcome:<br>• 1st intervention & 2nd intervention: not successful<br>• 3rd intervention: Successful- decrease in aggression with situational time out helped him calm down and return to demands of hygiene opportunity to calm down; less intrusive procedures; allowed for verbal prompting to be achieved;	
**Swan, L & Alderman, N., 2003**<br><br>**United Kingdom**	Case report<br><br>To determine what treatment approaches might be helpful in reducing aggressive behaviour in TBI survivors utilizes neurobehavioral expectations scale	N = 3<br><br>Age: 23, 27, 43<br><br>Sex/Gender [not specified]*: All men<br><br>Race/ethnicity: NR<br><br>Other: N/A	N = 3 (100 %)<br><br>Definition: NR<br><br>Method of identification: CT scan, GCS score<br><br>Severity: NR<br><br>Time since injury: NR	N = 1 (33.3 %)<br><br>Assault	Intervention: Neurobehavioral programme<br><br>Team:<br>• Occupational therapist<br>• Medical consultants<br>• Speech language pathologists<br>• Teachers<br>• Physiotherapists<br>• Nursing staff<br><br>Outcome:<br>• Greater control of behaviours<br>• No physical aggression at 2 year follow up	Barrier: Aggressive behavior<br><br>Facilitator: Carbamazepine in conjunction with the neurobehavioural programme may have had a role to play in the management of KA's aggression<br><br>Gap: NR

Supplementary File 2

Charting Table

Study (Author, Year, Country)	Study Design & Objective	Study Sample	TBI Status	Violence Status	Rehabilitation Intervention, Team, Outcome	TBI-Specific Barriers, Facilitators, and Gaps
**Ta'eed et al., 2015**<br><br>**Australia**	Quantitative<br><br>To look at services accessed by those with mild injuries who have no physical disabilities (hypothesis that increased injury severity associated with referral to more disciplines)	N = 175<br><br>Age (mean ± SD): 37 ± 15.12; age range 16-87 years<br><br>Gender: 68 % male<br><br>Race/ethnicity: NR<br><br>Other: Average of 10.78 years of education	N=175 (52 % mild, 31 % moderate, 17 % severe)<br><br>Definition: NR<br><br>Method of identification: Diagnosis of TBI (not specified)<br><br>Severity: 52 % mild, 31 % moderate, 17 % severe<br><br>Time since injury: NR	n=68 (39 % sustained TBI through assault-related injury<br><br>Assault	Intervention: Public post acute rehabilitation unit<br><br>Team:<br>• Physical therapist<br>• Occupational therapist<br>• Psychologist<br>• Social worker<br>• Nurse<br>• Speech language pathologist<br>• Dietician<br><br>Outcome: NR	Barrier:<br>• Problems experienced by mild TBI are intangible and easily overlooked by medical services<br>• Symptoms of anxiety overlooked or assumed normal reactions to trauma less likely refer to psychology<br><br>Facilitator: NR<br><br>Gap:<br>• Milder TBI may receive less therapy overall - complete picture would obtain by measuring amount of therapy participants received in each discipline<br>• Referred to services by research staff not typical rehab pathways
**Tate et al., 2005**<br><br>**Australia**	Quantitative<br><br>• To characterise service utilisation in the entire series of 467 people with TBI, all of whom were currently in receipt of services from the community team during the calendar year 2000"<br>• To examine changes over time, specifically in terms of therapy interventions, by examining the aforementioned	N = 50<br><br>Age range: 15 to 78 years (average 34.7)<br><br>Sex: 81 % male<br><br>Race/ethnicity: NR<br><br>Other: Psychosocial functioning was disrupted for many individuals: in the occupational domain, for instance, whereas pretrauma 88 % were employed (52 % full-time and 12 % part-time) or were students (24 %); post trauma only 28 % were	N=50, however, unclear if all had TBI<br><br>Definition: Community service sustained TBI<br><br>Method of identification: NR<br><br>Severity: NR<br><br>Time since injury:<br>• Median time post-trauma was 28 months<br>• 57 % sustained injury in preceding three years	13 % sustained TBI through assault<br><br>Assault	Intervention: Community TBI service<br><br>Team:<br>• Case managers<br>• Physiotherapist<br>• Occupational therapist<br>• Social worker<br>• Clinical psychologist<br>• Medical clinic<br>• Speech pathologist<br>• Neuropsychologist<br><br>Outcome: NR	Barrier: NR<br><br>Facilitator: NR<br><br>Gap:<br>• Sample is small & unbalanced<br>• Lack of data related to everyday functional tasks<br>• Other studies have larger sample and measures of instrumental activities daily living

Supplementary File 2

Charting Table

Study (Author, Year, Country)	Study Design & Objective	Study Sample	TBI Status	Violence Status	Rehabilitation Intervention, Team, Outcome	TBI-Specific Barriers, Facilitators, and Gaps
	random sample of the larger group and comparing the interventions received during 2000 with those received two years leter, during 2002.	employed (8 % full-time and 14 % part-time) or were students (6 %)				
**Voelbel et al., 2021**<br><br>**United States**	RCT<br><br>This study investigated the effects of a neuroplasticity-based computerized cognitive remediation program for auditory information processing in adults with a chronic TBI	N = 48<br>Intervention: N – 20<br>Control: N - 28<br><br><u>Age (mean):</u><br>• Intervention 44.60 years<br>• Control 44.50 years<br><br><u>Sex(n%):</u><br>Biological sex:<br>Intervention (n =20)<br>• Males 13(65 %)<br>• Females 7(35 %)<br>Control (n = 28)<br>• Males 12(43 %)<br>• Females 16(57 %)<br><br><u>Ethnicity (n%):</u>	N= 48 (100 %)<br><br><u>Definition:</u> NR<br><br><u>Method of identification:</u> NR<br><br><u>Severity</u>: mild n=15; moderate n=5; severe n=28<br><br><u>Time since injury:</u> TBI experienced 1- 38 years prior to enrollment in study	Intervention: N=3 assault (16 %);<br>Control: N=3 assault (11 %)<br><br>Assault	<u>Intervention:</u> Brain Fitness Program or Focus on Auditory Processing Program<br><br><u>Team</u>: NR<br><br><u>Outcome:</u> The intervention group improved on objective neuropsychological measures and a self-report measure of subjective functioning compared to the control group.	<u>Barrier:</u> NR<br><br><u>Facilitator:</u> NR<br><br><u>Gap:</u><br>• Sample is small & unbalanced<br>• Lack of data related to everyday functional tasks<br>• Other studies have larger sample and measures of instrumental activities daily living

Supplementary File 2

Charting Table

Study (Author, Year, Country)	Study Design & Objective	Study Sample	TBI Status	Violence Status	Rehabilitation Intervention, Team, Outcome	TBI-Specific Barriers, Facilitators, and Gaps
		Intervention (n=20):<br>Caucasian 13(65 %)<br>Hispanic 6(30 %)<br>African American 0(0 %)<br>Asian-American 1(5 %)<br><br>Control (n = 28)<br>Caucasian 20(71 %)<br>Hispanic 4(14 %)<br>African American 3(11 %)<br>Asian American 1(4 %)<br><br>Other:<br>Mean Education years:<br>Intervention 15.5<br>Control 16.14.				
**Watson et al., 2009**	Case study<br><br>Successful demonstration of reduction in aggressive behaviour in a survivor 10 years post injury in a residential neurorehab unit	N = 1<br><br>Age: Mid-twenties<br><br>Sex/Gender [not specified]*: Male<br><br>Race/ethnicity: NR<br><br>Other: Musician	N = 1 (100 %)<br><br>Definition: NR<br><br>Method of identification: NR<br><br>Severity: NR<br><br>Time since injury: Mid-twenties when he sustained brain injury	N = 1 (100 %)<br><br>Penetrating brain injury resulting from gunshot wound	Intervention: Specialist neurobehavioral unit<br><br>Team:<br>• Physical therapist<br>• Occupational therapist<br>• Speech language pathologist<br>• Consultant clinical neuropsychologist<br>• Consultant neuropsychiatrist<br>• Graduate psychologist<br><br>Outcome:<br><br>Decrease in aggressive behaviour from start of program	Barrier:<br>• Physical environment - a lot of structure needed<br>• Insufficient staff<br>• Lack of training<br><br>Facilitator:<br>• Utilizing the programme within a neuro rehabilitation unit that specialized in specific behavioural service<br>• Increased positive interaction between staff and survivor<br>• Regular feedback<br><br>Gap: NR

Supplementary File 2

Charting Table

Study (Author, Year, Country)	Study Design & Objective	Study Sample	TBI Status	Violence Status	Rehabilitation Intervention, Team, Outcome	TBI-Specific Barriers, Facilitators, and Gaps
**Wertheimer et al., 2008**<br><br>**United States**	RCT<br><br>To examine the functional status of persons surviving a severe penetrating traumatic brain injury (TBI) resulting from a gunshot would who require inpatient rehabilitation	N = 90<br><br>Age (mean): 32.4 in gunshot groups; 31.8 in MVC participant groups<br><br>Sex: 82 % Male in gunshot groups; 80 % male in MVC participant groups<br><br>Race:<br>Gunshot wound group: White (7 %); African American (91 %); Hispanic (0 %) & other (2 %)<br><br>MVC group: white (47 %); African American (49 %), Hispanic (4 %), other (0%)<br><br>Other:<br>Demographics & n(%)<br>Gunshot Group:<br>Education<br>• 1 through 8 (17 %)<br>• 9 through 11 (38 %)<br>• GED (2 %)<br>• High School (31 %)<br>• Trade School (0 %)<br>• Some college (12 %) and associate's degree (0 %)<br><br>Marital Status:<br>• Single (69 %)<br>• Married (11 %)<br>• Divorced (7 %)	N = 90 (100 %) sustained TBI through violence or MVC.<br><br>Definition: NR<br><br>Method of identification: NR<br><br>Severity: Severe brain injury (penetrating and nonpenetrating)<br><br>Time since injury: NR	N = 45 (50 %) sustained TBI through violence<br><br>Gunshot wound	Intervention: Rehabilitation hospital within a TBI model system<br><br>Team: NR<br><br>Outcome: NR	Barrier:<br><br>Facilitator:<br><br>Gap:<br>• Did not look at survivors who sustained gunshot wounds who went to other treatment settings (skilled nursing homes)<br>• Investigate magnitude of challenge and quality of life for care providers and/or family members

Supplementary File 2

Charting Table

Study (Author, Year, Country)	Study Design & Objective	Study Sample	TBI Status	Violence Status	Rehabilitation Intervention, Team, Outcome	TBI-Specific Barriers, Facilitators, and Gaps
		• Separated (4 %)<br>• Widowed (9 %)<br><br>Employment status (premorbid):<br>• Employed (40 %)<br>• Unemployed (38 %)<br>• Full-time student (11 %)<br>• Homemaker (0 %)<br>• Retired (4 %)				
**Wong et al., 1997**<br><br>**United States**	Case study<br><br>To identify, "the nutrition and medical challenges of maintaining adequate maternal and fetal health in a pregnant comatose patient	N = 1<br><br>Age: 34<br><br>Sex: Female<br><br>Race/ethnicity: NR<br><br>Other: N/A	N=1 (100 %) sustained TBI through spousal abuse<br><br>Definition: closed head injury (was admitted in a coma); underwent craniotomy<br><br>Method of identification: CT scan of the head demonstrated a right frontal, temporal, and parietal acute subdural hematoma, with a right-to-left subfascial herniation.<br><br>Severity: NR<br><br>Time since injury: NR	N = 1(100 %)<br><br>Spousal abuse	Intervention: Occupational and physical therapy daily for range of motion and strengthening exercises for extremities and to prevent contractions<br><br>Team:<br>• Physical therapist<br>• Occupational therapist<br>• Nurse<br>• Nutrition<br>• Physicians<br><br>Outcome: NR	Barrier: NR<br><br>Facilitator: NR<br><br>Gap: NR

Supplementary File 2

Charting Table

Study (Author, Year, Country)	Study Design & Objective	Study Sample	TBI Status	Violence Status	Rehabilitation Intervention, Team, Outcome	TBI-Specific Barriers, Facilitators, and Gaps
**Wong et al., 2021**<br><br>**United States**	Cohort study<br><br>To describe the clinical and patient-reported outcomes for people with post-concussion symptoms after a protocol sequenced to address cervical dysfunction and benign paroxysmal positional vertigo within first three weeks of injury, followed by integrated vision and vestibular therapy	N = 38<br><br>Age (mean ± SD): 26.9 ± 19.7 years<br><br>Sex:<br>Male (n=25)<br>Female (n = 13)<br><br>Race/ethnicity: NR<br><br>Other: N/A	N=38 people with post concussion symptoms<br><br>Definition: People with post-concussion symptoms due to sports, falls, assaults and motor vehicle accident injuries<br><br>Method of identification: NR<br><br>Severity: NR<br><br>Time since injury: 31.6-50.4 post injury	N=3 (7.9 % sustained TBI through assault)<br><br>Assault	Intervention: Manual therapy integrated with vision and vestibular therapy<br><br>Team: Trained concussion clinic treatment specialist<br><br>Outcome:<br>• Significant improvements at post-test for general post concussion symptoms;<br>• cervical range of motion returned to normal<br>• Subjective vision symptoms improved<br>• Subjective patient-reported outcome improved significantly	Barrier: NR<br><br>Facilitator: NR<br><br>Gap: NR
**Yen et al., 2022**<br><br>**Taiwan**	Quantitative<br><br>To investigate the possible enhancing effects of revised progressive early mobilization on functional mobility and the rate of out-of-bed mobility attained by patients with moderate-to-severe TBI	N = 86<br><br>Age (mean ± SD): Control group (55.77 ± 15.78) & intervention group (55.23 ± 21.41)<br><br>Sex:<br>Male, control group (n=32), intervention group (n=30)<br><br>Race/ethnicity: NR<br><br>Other: NR	N = 86 (100 %)<br><br>Definition: NR<br><br>Method of identification: NR<br><br>Severity: Moderate to severe TBI<br><br>Time since injury: NR	Control group n=1 (2.3 %) & mobility intervention group n=1 (2.4 %) sustained TBI through violence<br><br>Violence	Intervention: Progressive mobilization protocol five times per week (one intervention every working day)<br><br>Team: NR<br><br>Outcome:<br>Patients in intervention group at discharged moved to level 1, 2, or 3 on Modified ICU Mobility Scale, while patients in control group remained at Level 0; 100 % of intervention target of sitting in bed, 57% achieved out-of-bed early mobilization target of	Barrier: NR<br><br>Facilitator: NR<br><br>Gap:<br>• Lack of support for functional recovery following progressive early mobilization protocol for trauma patients<br>• No gold standard for progressive early mobilization protocol

Supplementary File 2

Charting Table

Study (Author, Year, Country)	Study Design & Objective	Study Sample	TBI Status	Violence Status	Rehabilitation Intervention, Team, Outcome	TBI-Specific Barriers, Facilitators, and Gaps
					level 3 activity (sitting on edge of bed).	

Notes:
[a]Study sample's characteristics are reported as described in the manuscript; for example, if the study used the term "gender" but described their participants as "males" or "females", this was reported as "Gender [Males]" in the table.

*Article did not specify if they collected sex or gender

**Article did not specify if they collected race or ethnicity

AIS: Abbreviated Injury Score; **ASMT:** Anger Self Management Training; **DSM-5:** Diagnostic and Statistical Manual of Mental Disorders, Fifth Edition; **EEG:** Electroencephalogram; **EF:** Executive Functioning; FIM**:** Functional Independence Measure; **IQ:** Intelligence quotient; **LOC:** Loss of Consciousness; **MEG**: Magneto-encephalography; **MVC:** Motor Vehicle Collision; **NDT:** Neurodevelopmental Treatment; **NR:** Not Reported; **PT:** Physiotherapy; **PTA:** Post-Traumatic Amnesia; **PTSD:** Post-traumatic Stress Disorder; **SMART-CPT:** A hybrid Cognitive Processing Therapy; **TAU:** Treatment as usual**; TBI:** Traumatic Brain Injury; **TLNS:** Translingual neurostimulation; **TROI:** Two-step Resilience-Oriented Intervention;
